# Crystalline 3D covalent organic frameworks with nbo topology

**DOI:** 10.1126/sciadv.aeg6230

**Published:** 2026-07-10

**Authors:** Soshi Hirota, Haruki Sugiyama, Nao Hirata, Sachiko Nakano, Junichi Usuba, Yuh Hijikata, Ryotaro Matsuda, Takanori Nakane, Akihiro Kawamoto, Genji Kurisu, Yasutomo Segawa

**Affiliations:** ^1^ Institute for Molecular Science, Myodaiji, Okazaki, 444-8787, Japan.; ^2^ The Graduate University for Advanced Studies, SOKENDAI, Myodaiji, Okazaki, Japan.; ^3^ Neutron Industrial Application Promotion Center, Comprehensive Research Organization for Science and Society, Tokai, Ibaraki 319-1106, Japan.; ^4^ Research Center for Net Zero Carbon Society, Institute of Innovation for Future Society, Nagoya University, Nagoya, Japan.; ^5^ Department of Chemistry and Biotechnology, School of Engineering, and Department of Materials Chemistry, Graduate School of Engineering, Nagoya University, Chikusa-ku, Nagoya, Japan.; ^6^ Institute for Protein Research, Osaka University, 3-2 Yamadaoka, Suita, Osaka 565-0871, Japan.; ^7^ JEOL YOKOGUSHI Research Alliance Laboratories, Graduate School of Frontier Biosciences, Osaka University, 1-3 Yamadaoka, Suita, Osaka 565-0871, Japan.; ^8^ Department of Macromolecular Science, Graduate School of Science, Osaka University, Toyonaka 560-0043, Japan.; ^9^ Institute for Open and Transdisciplinary Research Initiatives, Osaka University, 2-1 Yamadaoka, Suita, Osaka 565-0871, Japan.

## Abstract

Three-dimensional covalent organic frameworks (3D COFs) are promising crystalline porous materials, but the elucidation of their structure remains challenging, particularly for those featuring spiroborate linkages. Herein, we report the synthesis of a 3D crystalline COF with nbo topology, constructed from a rigid square-planar monomer, tetracyclopentatetraphenylene (TCTP), and spiroborate linkages. Theoretical calculations revealed that the TCTP core has higher rigidity than phthalocyanine, effectively suppressing structural fluctuations during framework formation. The structure of the resulting **TCTP-COF** was successfully determined using microcrystal electron diffraction (MicroED), revealing a noninterpenetrated cubic framework. **TCTP-COF** exhibits high crystallinity, thermal stability up to 320°C, and permanent porosity with a Brunauer-Emmett-Teller surface area of 1360 square meters per gram. This work represents the structural determination of a spiroborate-linked 3D crystalline COF using MicroED methods, providing a design strategy for expanding the chemical space of highly ordered 3D COF architectures.

## Teaser

A crystalline spiroborate-linked 3D framework with nbo topology was synthesized, and its structure was determined by MicroED.

## INTRODUCTION

Three-dimensional covalent organic frameworks (3D COFs) are crystalline porous polymers with covalently linked organic units that exhibit well-defined topologies. Since the first 3D COF was reported by Yaghi and co-workers in 2017 (*1*–*5*), these materials have attracted considerable attention owing to their periodic order, large surface areas, and the ability to tune their structures and functions at the molecular level. Despite these advantages, synthetic methods used to produce COFs often result in amorphous or poorly crystalline solids, and obtaining highly ordered frameworks in a reliable manner remains challenging. As a result, atomic-level structural determination has been achieved for only a limited number of 3D COFs, leaving the relationships between structure and properties poorly understood (*6*–*9*).

The development of crystalline COFs whose structures can be determined via single-crystal x-ray diffraction (SCXRD) or microcrystal electron diffraction (MicroED) has been important for the advancement of COF chemistry (*10*–*12*). In 2013, the first SCXRD analysis of a nonporous 3D crystalline COF based on highly reversible azodioxy linkages was reported. This was then followed by the elucidation of the first imine-linked porous 3D crystalline COF structure by Wang, Sun, Yaghi and co-workers using SCXRD (*13*, *14*). More recently, 3D crystalline COFs have demonstrated superior performance to polycrystalline COFs in a diverse array of applications, including molecular separations, catalysis, and thermal conduction, thus highlighting how important the precise understanding of the COF structure is for the control of functional properties (*15*–*18*). However, most crystalline COFs reported to date rely on imine linkages, and hence, their structural diversity remains limited. Thus, exploring the modes of bond formation for crystalline COF synthesis represents a promising strategy to expand the chemical space of COFs and uncover novel structure-function relationships.

**Fig. 1.**
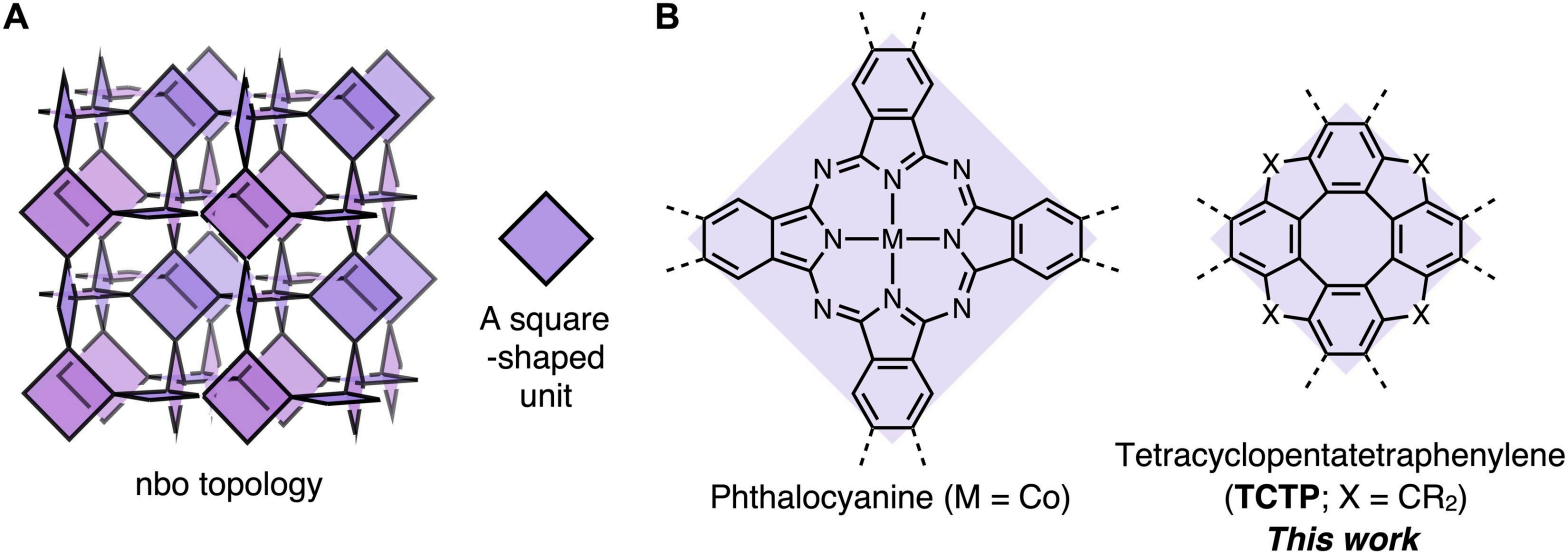
3D COFs with nbo topology and their structural units. (**A**) The nbo topology. (**B**) Square units used to create nbo COFs.

In this study, we focused on borate anions as the linkage motif for constructing 3D crystalline COFs. Borates are known to form tetracoordinate spiro-type structures that are rigid and stable. A previous report of crystalline one-dimensional polymers featuring borate-based linkages further suggests that this motif could be extended to the construction of highly crystalline 3D COFs (*19*, *20*). Although several 3D COFs using borate linkages have been reported, it has not yet been possible to conduct a single-crystal structural analysis on these COFs (*21*–*24*). For example, Chen, Ward, Cooper and co-workers (*25*–*27*) synthesized a 3D COF with nbo topology, which had limited crystallinity, by joining square-shaped Co-phthalocyanine units together through spiroborate linkages (Fig. 1, A and B).

Herein, we report the synthesis of a 3D crystalline COF constructed from tetracyclopentatetraphenylene (TCTP) (*28*–*30*), a hetero[8]circulene analog (*31*–*33*), and the successful determination of its structure using MicroED. Density functional theory (DFT) calculations reveal that the TCTP exhibits higher rigidity than phthalocyanine, identifying it as a suitable unit for suppressing structural fluctuations in crystalline COFs. The introduction of eight ethyl substituents onto the TCTP effectively hindered π-π stacking and imparted high solubility. The reaction of the monomer with trimethylborate afforded a crystalline COF, and MicroED measurements confirmed that the resulting COF adopts an ideal cubic framework with nbo topology. Powder x-ray diffraction (PXRD) and solid-state ^13^C nuclear magnetic resonance (NMR) spectroscopy studies indicated that the COF has high crystallinity and structural homogeneity, while gas adsorption measurements revealed its permanent porosity.

## RESULTS

**Fig. 2.**
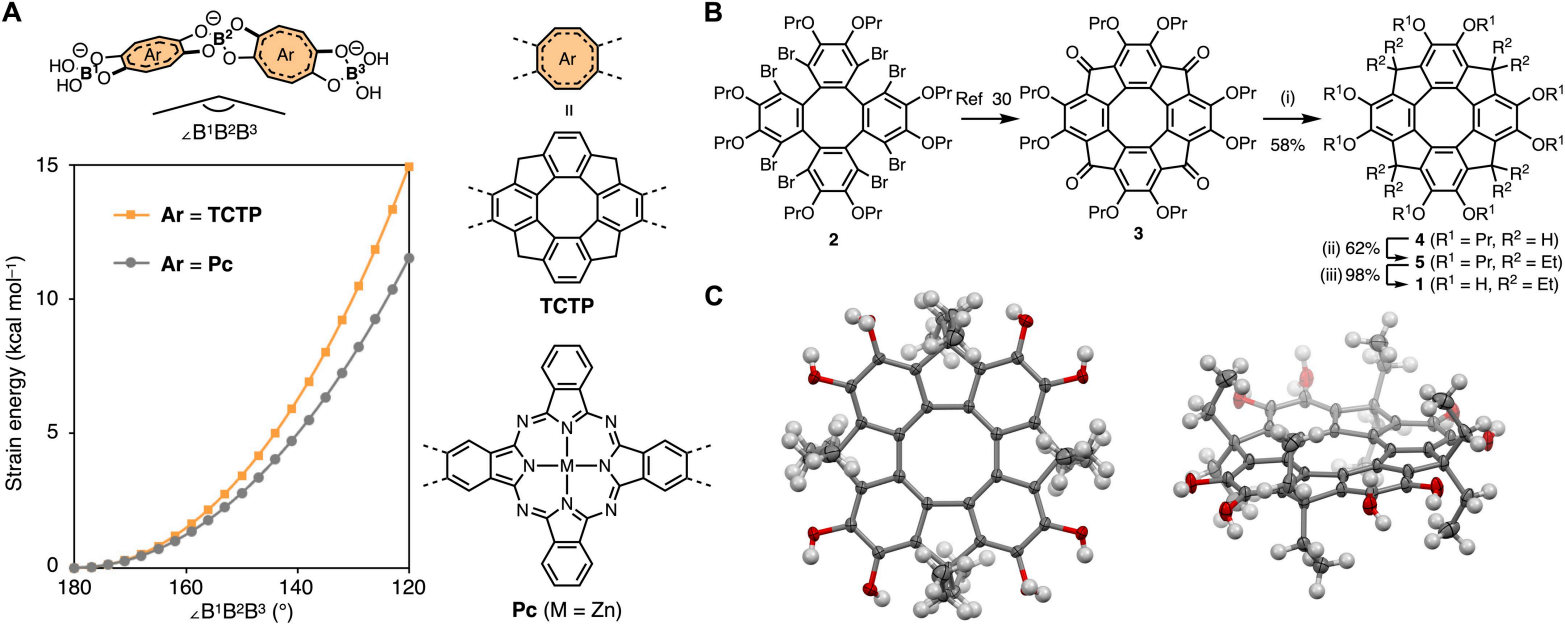
Design, synthesis, and structure of the square monomer unit. (**A**) Strain energy analysis of the borate depending on the ∠B^1^B^2^B^3^ angle calculated at the B3LYP level of theory with the LANL2DZ basis set for Zn and the 6-31G(d) basis set for other atoms. (**B**) Synthesis of 1 (*30*). Reaction conditions: (i) B_2_(OH)_4_, Pd/C, tetrahydrofuran (THF), reflux, 13 hours. (ii) Iodoethane, potassium *tert*-butoxide, THF, 19 hours. (iii) BBr_3_, CH_2_Cl_2_, reflux, 21 hours. Pr, *n*-propyl. (**C**) X-ray crystal structure of **1** at 50% thermal probability.

First, a square-planar molecular unit for the construction of the 3D COF was designed. Hetero[8]circulenes (Fig. 1B) are aromatic molecules in which the C═C moieties of the [8]circulene are replaced by heteroatoms. Depending on the incorporated elements, the hetero[8]circulenes adopt either planar (X = N, O, S, and C) or saddle-shaped (X = Si, Ge, and Se) structures. In this study, we selected TCTP (X = CR_2_) to achieve both a square-planar structure and a monomer unit with sufficient solubility. To evaluate the rigidity of the TCTP skeleton relative to that of phthalocyanine (**Pc**), we performed DFT calculations. As shown in Fig. 2A, we used spiroborate-bridged dimers to represent a simplified 3D COF substructure in our model. We varied the ∠B^1^B^2^B^3^ angles of the dimer over a range from 180° to 120° and computed their relative strain energies. The strain energy of the spiroborate-bridged **TCTP** dimer is ~30% higher than that of the **Pc** dimer (14.9 kcal mol^−1^ for **TCTP** versus 11.5 kcal mol^−1^ for **Pc** at 120°). This result indicates that the TCTP-based units are less prone to bending during the formation of the spiroborate-bridged 3D COFs, suggesting that the TCTP-based 3D COFs would be formed with fewer structural defects.

Following the molecular design described above, we synthesized TCTP derivative **1** as the monomer unit to be used for construction of the 3D COF, and the synthetic route is shown in Fig. 2B. The preparation of compound **2** was achieved by sequential bromination and carbonylation of octapropoxytetraphenylene (*30*). Starting from compound **3**, reduction of the carbonyl groups with tetrahydroxydiboron and palladium on carbon (Pd/C) afforded the TCTP derivative **4**. Subsequent base-promoted ethylation with iodoethane followed by deprotection of the propyl groups with boron tribromide yielded the desired monomer **1**. The structure of **1** was determined via SCXRD analysis and revealed that the TCTP core adopts a planar conformation and that the eight ethyl groups are oriented above and below the π plane, effectively suppressing π-π stacking (Fig. 2C).

**Fig. 3.**
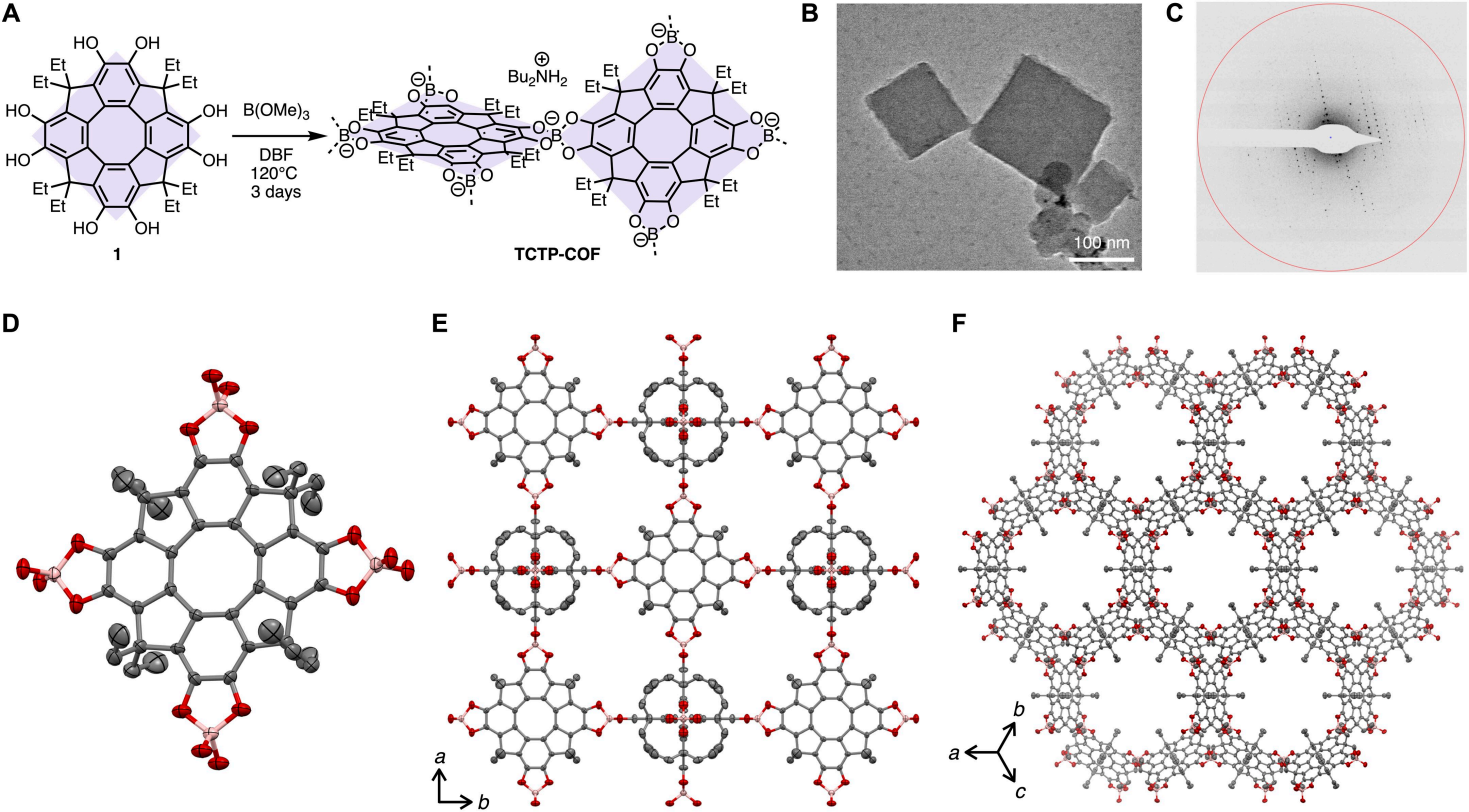
Synthesis and structure of TCTP-COF. (**A**) Synthesis of **TCTP-COF**. (**B**) TEM image of **TCTP-COF**. (**C**) Electron diffraction pattern of **TCTP-COF**. The red ring is drawn at ~0.8 Å. (**D** to **F**) Crystal structure of **TCTP-COF** determined using MicroED with thermal ellipsoids set at 50% where hydrogen atoms were omitted for clarity: (D) A TCTP moiety in **TCTP-COF**; (E and F) packing mode of **TCTP-COF**.

The synthesis of a 3D COF using **1** as the monomer was then performed. After screening, we found that a crystalline insoluble solid was produced following the reaction with trimethylborate in *N*,*N*-dibutylformamide (DBF) at 120°C for 3 days (Fig. 3A). The resulting solid consisted of crystalline particles (see fig. S8) with a maximum particle size of ~1 μm, which was insufficient for SCXRD analysis. Transmission electron microscopy (TEM) observations revealed that the small crystals of ~100 nm in diameter were square shaped (Fig. 3B).

To determine the structure of the COF, was subjected the solid to structural analysis by MicroED (Fig. 3C). As shown in Fig. 3 (D to F), the structure of the obtained crystals was clearly determined to be a cubic 3D COF with nbo topology (**TCTP-COF**), while the space group was *Im*-3*m* and the cell length had dimensions of *a* = 25.28 Å. The structure of **TCTP-COF** was found not to have any interpenetration. The ethyl groups on **TCTP** were observed, while the countercations (*n*-Bu_2_NH_2_^+^) were not observed, probably because of the disorder in the highly symmetric space group of **TCTP-COF**. Sharp diffraction spots extending to high resolution observed in MicroED indicates that **TCTP-COF** maintains high crystallinity even under the high vacuum used in TEM. The high-crystallinity **TCTP-COF** was probably due to the high rigidity and solubility of the TCTP unit as well as the reversibility of the spiroborate formation reaction.

**Fig. 4.**
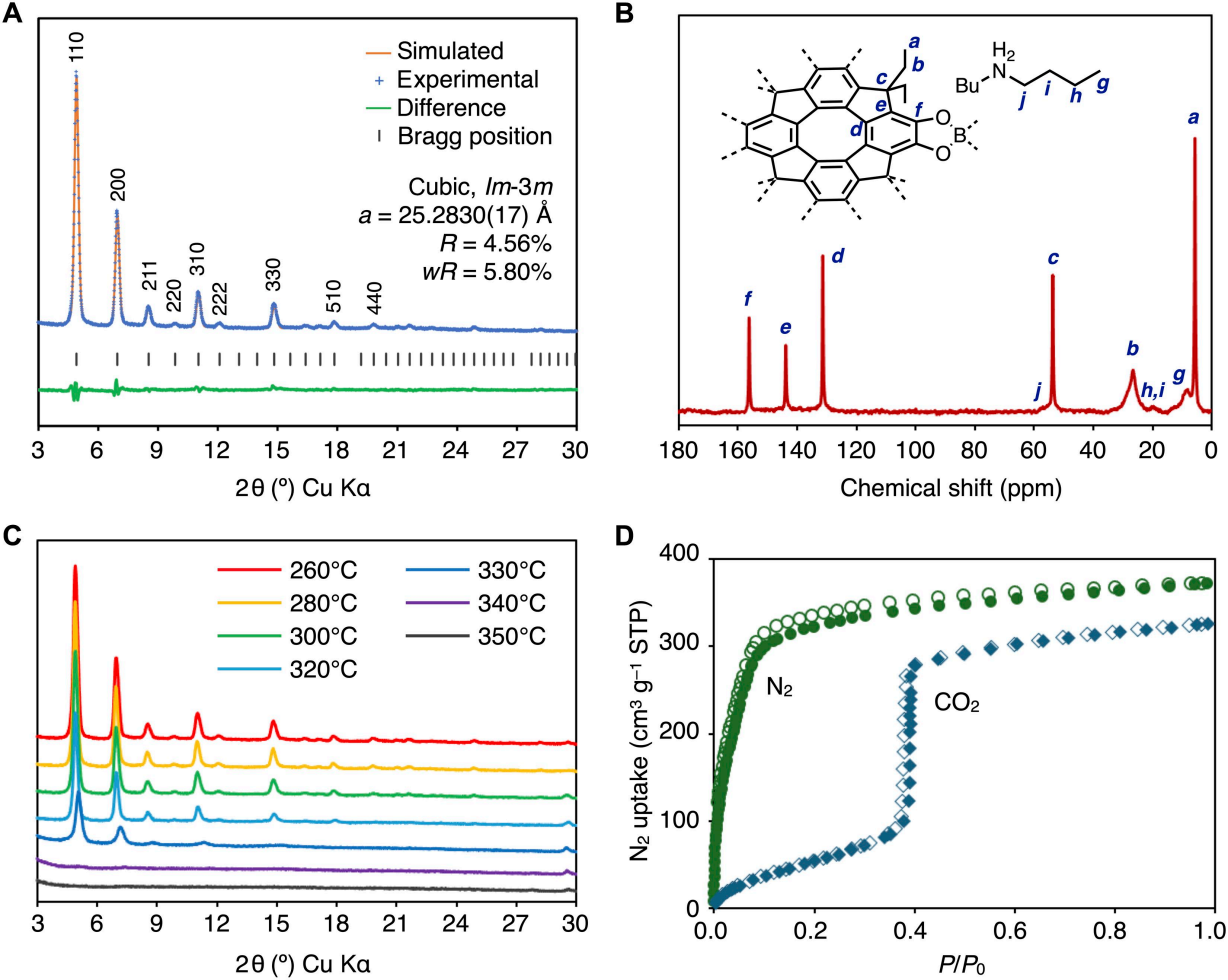
Properties of TCTP-COF. (**A**) PXRD pattern and Rietveld refinement of **TCTP-COF**. (**B**) ^13^C CP/MAS NMR spectrum of **TCTP-COF**. (**C**) PXRD patterns of **TCTP-COF** after heating at each temperature for 10 min. (**D**) N_2_ adsorption-desorption isotherm at 77 K and the CO_2_ adsorption-desorption isotherm at 195 K of **TCTP-COF**. Closed and open circles represent the adsorption and desorption isotherms, respectively.

The structure of **TCTP-COF** was analyzed in the bulk powder using PXRD. Diffraction using Cu Kα x-ray radiation yielded very narrow diffraction patterns, with the peaks at 4.92°, 6.98°, 8.57°, 9.83°, 11.05°, 12.13°, 14.87°, 16.37°, 17.87°, and 19.87° (Fig. 4A). The Rietveld refinement starting from the structure obtained using MicroED could be fitted nicely [*a* = 25.2830(*17*) Å, *wR* = 5.80%] and confirmed that the PXRD peaks correspond to the 110, 200, 211, 220, 310, 222, 330, 332, 510, and 440 planes, respectively.

The chemical properties of **TCTP-COF** were analyzed via Fourier transform infrared (FTIR) spectroscopy, solid-state ^13^C cross-polarization/magic-angle spinning nuclear magnetic resonance (^13^C CP/MAS NMR) spectroscopy, and ^11^B magic-angle spinning nuclear magnetic resonance (^11^B MAS NMR) spectroscopy. Relative to the FTIR spectrum of monomer **1**, the **TCTP-COF** spectrum showed absorption bands corresponding to the spiroborate linkages and the loss of absorption bands corresponding to hydroxyl groups (fig. S10). The solid-state ^13^C NMR signals showed very narrow peaks demonstrating the high homogeneity of **TCTP-COF** (Fig. 4B). While all peaks corresponding to the TCTP core (156.4, 143.9, 131.5, and 53.8 ppm) and ethyl groups (26.6, and 5.8 ppm) were clearly identifiable, broadened peaks of the countercation (*n*-Bu_2_NH_2_^+^) were observed in the range of 30.0 to 20.0 ppm and 10.0 to 5.0 ppm, which is likely due to disordering. A very narrow peak was also observed at 18.6 ppm in the solid-state ^11^B NMR spectrum (fig. S13).

Next, the thermal stability and porosity of **TCTP-COF** were investigated. The synthesized **TCTP-COF** was heated from room temperature to 360°C under a nitrogen atmosphere and was then held at each temperature for 10 min before being analyzed at room temperature using PXRD. This measurement revealed that the PXRD pattern of the sample heated to 320°C remained almost unchanged (Fig. 4C). Thermogravimetric analysis revealed a two-stage weight loss (fig. S15). A weight loss of ~10% was observed below 100°C, followed by a further 20% weight loss at >320°C. The first loss was assigned to desorption of the solvent within the porous structure, while the second loss was due to degradation of the framework. Therefore, **TCTP-COF** was found to have thermal stability at temperatures above 300°C.

The porosity of **TCTP-COF** was evaluated through N_2_ and CO_2_ adsorption measurements at 77 and 195 K, respectively. Guest removal from **TCTP-COF** was achieved by vacuum heating. N_2_ adsorption isotherms at 77 K showed a rapid absorption at low relative pressures, characteristic of microporous materials. CO_2_ adsorption gradually increased up to a relative pressure of 0.4, followed by an increase thereafter (Fig. 4D). The presence of small hysteresis and steps in both CO_2_ and N_2_ represent typical adsorption behavior observed in pores of an intermediate size between micropores and mesopores (*34*). The Brunauer-Emmett-Teller and Langmuir surface areas were found to be 1360 and 1620 m^2^ g^−1^, respectively, which correspond to 36 and 43% of the N_2_-accessible surface area calculated on the basis of the **TCTP-COF** framework without countercations. Furthermore, the synthesized **TCTP-COF** maintained its crystallinity and high gas adsorption capacity, even after complete guest removal. These results suggest that **TCTP-COF** has persistent porosity.

## DISCUSSION

In summary, we have successfully synthesized a borate-linked 3D crystalline COF with nbo topology, **TCTP-COF**, by using a highly rigid TCTP monomer. The rational molecular design, which incorporated ethyl substituents to prevent π-π stacking and enhance solubility, was crucial for achieving high crystallinity. The structure was unambiguously elucidated using MicroED analysis, confirming the presence of an ideal cubic framework without interpenetration. **TCTP-COF** demonstrates excellent structural homogeneity, high thermal stability, and permanent microporosity. This study not only reports the structural elucidation of a borate-linked 3D crystalline COF via electron diffraction but also demonstrates the potential of hetero[8]circulene analogs as robust building blocks for complex 3D frameworks. These findings pave the way for both the precise construction of functional ionic COFs and the exploration of their structure-property relationships to enable their use in advanced applications.

## MATERIALS AND METHODS

### Materials

Unless otherwise noted, all materials were obtained from commercial suppliers and used without further purification. Tetrahydrofuran and Et_2_O for reactions were purified by passing through a solvent purification system (Glass Contour). All reactions were performed with dry solvents under an atmosphere of nitrogen in dried glassware with standard vacuum-line techniques. Work-up and purification procedures were carried out with reagent-grade solvents under air for compounds **4** and **5** or under N_2_ atmosphere for compound **1**. Tetracarbonylated TCTP **3** (*30*) was synthesized according to reported procedure.

### Synthesis of TCTP-COF

To a 6-ml glass tube were added **1** (19.5 mg, 28.4 μmol), B(OMe)_3_ (6.3 μl, 57 μmol), and DBF (0.93 ml) in an argon-filled glove box. The mixture was degassed by freeze-pump-thaw cycling and sealed under vacuum using a Schlenk line and oil pump. After warming to room temperature, the reaction tube was put into an oven at 120°C for 3 days. After cooling to ambient temperature, the crude product was washed with hexane, EtOAc, water, CHCl_3_, and Et_2_O and then dried in vacuo to afford **TCTP-COF** as a pale blue crystalline solid (16.5 mg, 61%).

## Supplementary Materials

### The PDF file includes:

Supplementary Materials and Methods Legend for data S1 Experimental Section Figs. S1 to S21 Tables S1 to S5 References
