# Supplementary material for: Crystalline 3D covalent organic frameworks with nbo topology
Source: Sci Adv. 2026 Jul 10;12(28):eaeg6230. doi: 10.1126/sciadv.aeg6230 (PMC13353416; doi:10.1126/sciadv.aeg6230)
Supplement: Supplementary file 1 — Supplementary Materials and Methods Legend for data S1 Experimental Section Figs. S1 to S21 Tables S1 to S5 References [file sciadv.aeg6230_sm.pdf]

Science Advances
AAAS

Supplementary Materials for

Crystalline 3D covalent organic frameworks with nbo topology

Soshi Hirota *et al.*

Corresponding author: Yasutomo Segawa, segawa@ims.ac.jp

Sci. Adv. **12**, eaeg6230 (2026)
DOI: 10.1126/sciadv.aeg6230

The PDF file includes:

Supplementary Materials and Methods
Legend for data S1
Experimental Section
Figs. S1 to S21
Tables S1 to S5
References

Other Supplementary Material for this manuscript includes the following:

Data S1

1. Materials and methods

Synthesis and purification

Unless otherwise noted, all materials were obtained from commercial suppliers and used without further purification. Tetrahydrofuran (THF) and Et_2O for reactions were purified by passing through a solvent purification system (Glass Contour). All reactions were performed with dry solvents under an atmosphere of nitrogen in dried glassware with standard vacuum-line techniques. Work-up and purification procedures were carried out with reagent-grade solvents under air for compounds **4** and **5** or under N_2 atmosphere for compound **1**. Tetracarbonylated tetracyclopentatetraphenylene **3** (*30*) was synthesized according to reported procedure. Analytical thin-layer chromatography (TLC) was performed using E. Merck silica gel 60 F254 precoated plates (0.25 mm). The developed chromatograms were analyzed by UV lamp (254 or 365 nm). Silica gel column chromatography was performed with KANTO Silica Gel 60N (spherical, neutral, 40-100 μm).

Measurements

The high-resolution mass spectra (HRMS) were obtained using a JEOL JMS-777V (FAB MS) with 3-nitrobenzyl alcohol for **4**, a Bruker Daltonics microflex LRF (MALDI-TOF MS) with 7,7,8,8-tetracyanoquinodimethane (TCNQ) for **5**, or a Bruker Daltonics maXis (ESI-TOF MS) for **1**.

Melting points were measured on a MPA100 Optimelt automated melting point system or not measured.

Nuclear magnetic resonance (NMR) spectra were recorded on a JEOL JNM-ECS400 (^{1}H 400 MHz, ^{13}C 100 MHz) spectrometer. Chemical shifts for ^{1}H NMR are expressed in parts per million (ppm) relative to $CHCl_3$ (δ 7.26 ppm). Chemical shifts for ^{13}C NMR are expressed in ppm relative to $CDCl_3$ (δ 77.16 ppm). Data are reported as follows: chemical shift, multiplicity (s = singlet, t = triplet, q = quartet, tq = triplet of quartet), coupling constant (Hz), and integration. NMR spectra of new products were shown in Figs. S16–S21.

X-ray crystallography was performed with a RIGAKU XtaLAB Synergy Custom system equipped with mirror monochromated Mo Kα radiation (λ = 0.71073 Å) and HyPix-6000HEIC detector for **4** and **1**, or a RIGAKU Supernova with micro-focus Cu Kα radiation (λ = 1.54184 Å) and EOS S2 detector for **5**. The suitable crystals were mounted with mineral oil on a MiTeGen MicroMounts and transferred to the kappa goniometer. Cell parameters were determined and refined, and raw frame data were integrated using CrysAlisPro (Rigaku Oxford Diffraction, 2018). The structures were solved by dual methods using SHELXT (*35*) and refined by full-matrix least-squares techniques against F^2 with SHELXL-2018/3 (*36*) using Olex2 software package (*37*). The intensities were corrected for Lorentz and polarization effects. All non-hydrogen atoms were refined with anisotropic displacement parameters. Hydrogen atoms were refined isotropically using the riding model. For the analysis of the crystal structure of **5**, the solvent mask method (*38*) implemented in Olex2 was applied because disordered solvent molecules could not be modeled (Fig. S6). Details of the crystal data and a summary of the intensity data collection parameters for **4**, **5**, and **1** are listed in Table S1.

MicroED datasets of **TCTP-COF** were acquired with a Talos Arctica (ThermoFisher Scientific) running at 200 kV. The microscope was controlled by SerialEM (*39-41*). Crystals were loaded on QuantiFoil Mo R0.6/1.0 grids. Crystals remained at the liquid nitrogen temperature in the scope. Crystals were thin plates with edges of a few hundred nanometers (Fig. S7). Continuous rotation diffraction patterns were recorded on a Falcon 3 direct electron detector in the integration mode (*42*) with a parallel illumination of a diameter of ~ 1.6 um at an electron flux of ~ 0.06 electron/Å^2/sec. Crystals were rotated for ~ 63° at ~ 0.95°/sec. Detector frames were binned by 2 (*i.e.* to 2048 x 2048 pixels, 28 um/px) and fractionated such that each fraction corresponded to ~ 0.435° of rotation. The virtual camera distance was ~ 615.5 mm. More than 1000 crystals from two grids were screened and rotation datasets from 439 crystals were recorded.

MicroED datasets were processed with the DIALS suite (*43,44*) according to our established protocol (*45*). GNU parallel (*46*) powered parallel processing. Out of the 439 measured crystals, 343 crystals were indexed in the unit cell corresponding to **TCTP-COF** (others failed in indexing or corresponded to contaminating inorganic borate salts). Crystals better than 0.89 Å (as judged by CC1/2 at 0.89Å > 0.30) were subjected to filtering and clustering analysis by xia2.multiplex (*47*), which selected 83 high-resolution isomorphic crystals. Intensities were scaled with dials scale (*48*). The merging statistics is in Table S3. The scaled intensities were phased by SHELXT and kinematically refined by SHELXL in the Olex2 GUI (*35-37*). All non-hydrogen atoms were refined with anisotropic displacement parameters. Hydrogen atoms were refined isotropically using the riding model. For the analysis of the crystal structure of **TCTP-COF**, the solvent mask method (*38*) implemented in Olex2 was applied because disordered countercations could not be modeled. Details of the crystal data and a summary of the intensity data collection parameters are listed in Table S2.

Powder X-ray diffraction (PXRD) data patterns of **TCTP-COF** were acquired using Rigaku MiniFlex 600-C diffraction system with 600 W X-ray tube (Cu Kα) and D/teX Ultra2 silicon strip detector. Samples were held on a Si crystal zero background sample holder. PXRD patterns were measured over the 2θ range 3–50° in 0.01° steps over 47 minutes (1°/min). Rietveld refinement was performed with GSASII program (Fig. S9). The structure data obtained from MicroED was used as the initial structure. Details were listed in Table S4.

Fourier-transform infrared spectroscopy (FT-IR) was performed from a JASCO FT/IR-460plus spectrometer in the ATR mode in air (Fig. S10).

Solid-state ^{13}C NMR spectra using cross polarization magic angle spinning (*49*) (CP-MAS) method were measured at 150 MHz with a JEOL JNM-ECZ600II spectrometer and 3.2 mm ^{1}H-X double resonance MAS probe. Spectra were acquired at a spin rate of 20 kHz ± 5 Hz. Carbon spectral referencing is relative to tetramethylsilane, carried out by setting the high frequency signal from an external sample of adamantane to 29.5 ppm. ^{1}H Heteronuclear decoupling during the detection period was achieved using two pulse phase modulation (TPPM) (*50*) with a ^{1}H RF field of 105 kHz. Initial magnetizations of rare nuclei were enhanced by using ^{1}H-^{13}C cross-polarization with 11% amplitude sweep of the ^{1}H spin locking field (Figs. S11,S12) (*51*). Solid-state ^{11}B NMR spectra using single pulse

decoupling method were measured at 193 MHz with a JEOL JNM-ECZ600II spectrometer and 3.2 mm ^{1}H-X double resonance MAS probe. Spectra were acquired at a spin rate of 20 kHz ± 5 Hz. Boron spectral referencing is relative to boron trifluoride etherate, carried out by setting the high frequency signal from an external sample of the saturated solution of boric acid to 19.49 ppm (Figs. S13,S14).

Gas adsorption measurement was conducted on a microtrac-MRB BELSORP-mini volumetric gas adsorption analyzer. **TCTP-COF** was activated under dynamic vacuum at 60 °C for 12 h prior to the measurement. Grade 1 N_2 and CO_2 gases were used (>99.99995 vol.% and >99.995 vol.%, respectively). The measurement temperature was controlled by a cryostatic temperature controller.

Thermogravimetric analysis (TGA) was carried out using a Rigaku TG-DTA8122. Sample was heated at a rate of 15 °C/min under a dry nitrogen gas flow (Fig. S15).

<u>Theoretical study</u>

The Gaussian 16 program (*52*) running on a NEC LX 110Rh system was used for optimization. Structures were optimized at B3LYP (*53,54*) level of theory and the basis sets of LANL2DZ for Zn or 6-31G(d) for others. The $\angle B^1B^2B^3$ angles of **TCTP** and **Pc** were restricted to the range of 120°–180°. Cartesian coordinates of optimized structures are included in Data S1 and the energies were listed in Table S5.

Data S1. Cartesian coordinates of optimized structures.

2. Experimental section

Synthesis of **4** (*55*)

To a 200-mL two-necked flask containing a magnetic stirring bar were added **3** (427 mg, 486 μmol), $B_2(OH)_4$ (888 mg, 9.91 mmol, 20 equiv.) and 10 w% of dry Pd/C (251 mg, 235 μmol, 0.48 equiv.). Dry THF (18.0 mL) was added and the reaction mixture was stirred at reflux in an oil bath for 13 h. After cooled to room temperature, the reaction mixture was passed through a plug of silica and Celite to remove black solids and washed with $CHCl_3$. The resulting mixture was evaporated to remove volatiles. The mixture was purified by silica gel column chromatography (CH_2Cl_2/hexane = 3:2) to afford **4** (230 mg, 58%) as yellow powder (Fig. S1). ^{1}H NMR (400 MHz, $CDCl_3$) δ 4.13 (t, J = 6.7 Hz, 16H), 3.82 (s, 8H), 1.86 (tq, J = 7.3 Hz, J = 6.7 Hz, 16H), 1.11 (t, J = 7.3 Hz, 24H); ^{13}C NMR (100 MHz, $CDCl_3$) δ 146.5 (4°), 137.9 (4°), 132.8 (4°), 74.7 (CH_2), 32.0 (CH_2), 23.9 (CH_2) 10.8 (CH_3); HRMS (FAB MS) *m*/*z*: $[M]^+$ calcd for $C_{52}H_{64}O_8$ 816.4596; found 816.4591. mp: 194.5–197.4 °C.

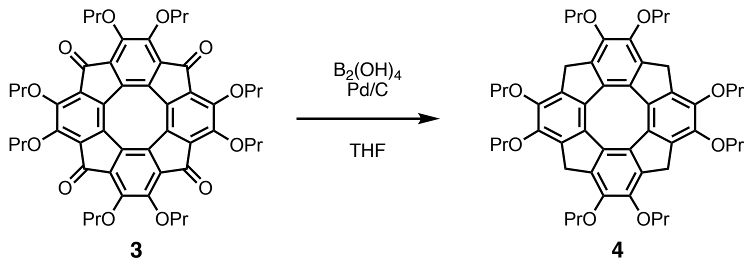

Fig. S1. The synthetic scheme of **4**.

Synthesis of **5**

To a two-necked test tube containing a magnetic stirring bar were added **4** (199 mg, 244 µmol) and ^{t}BuOK (549 mg, 4.90 mmol, 20 equiv.). Dry THF (1.0 mL) was added, and the resulting mixture was stirred at reflux in an oil bath for 1 h. Iodoethane (0.79 mL, 9.9 mmol, 40 equiv.) was added at 0 °C for 5 min. Dry THF (0.6 mL) was added and the reaction mixture was stirred at reflux in an oil bath for 19 h. After cooled to room temperature, the reaction mixture was quenched with water, extracted with $CHCl_3$, washed with brine, dried over Na_2SO_4, and then evaporated *in vacuo*. The mixture was purified by the column chromatography (hexane/CH_2Cl_2 = 3:1) to afford **5** as yellow powder (158 mg, 62%) (Fig. S2). ^{1}H NMR (400 MHz, $CDCl_3$) δ 3.99 (t, J = 7.0 Hz, 16H), 2.41 (q, J = 7.3Hz, 16H), 1.87 (tq, J = 7.3 Hz, J = 7.0 Hz, 16H), 1.10 (t, J = 7.3, 24H), 0.35 (t, J = 7.3 Hz, 24H); ^{13}C NMR (100 MHz, $CDCl_3$) δ 147.3 (4°), 142.4 (4°), 133.1 (4°), 72.7 (CH_2), 56.7 (4°), 28.2 (CH_2), 23.7 (CH_2), 10.9 (CH_3), 9.4 (CH_3); HRMS (MALDI-TOF MS) m/z: $[M]^+$ calcd for $C_{68}H_{96}O_8$ 1040.709971; found 1040.708960.

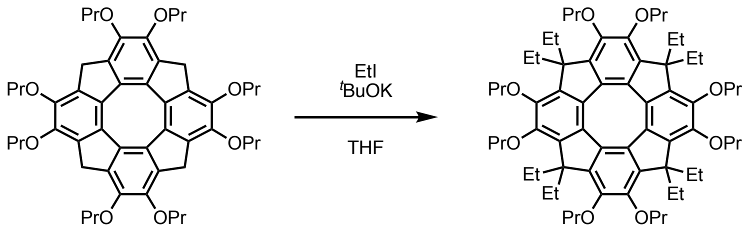

Fig. S2. The synthetic scheme of **5**.

Synthesis of **1**

To a two-necked test tube containing a magnetic stirring bar were added **5** (72.4 mg, 69.5 μmol) and dry CH_2Cl_2 (1.5 mL). The CH_2Cl_2 solution of BBr_3 (1.0 M, 2.1 mL, 2.1 mmol, 30 equiv.) was added at 0 °C, and the reaction mixture was stirred for 1 h and refluxed in an oil bath for 21 h. After cooled to room temperature, the reaction mixture was concentrated, and degassed water was added to the test tube and removing the supernatant by centrifugation. The insoluble solid was washed by degassed benzene and methanol, and then dried *in vacuo* to afford **1** as white powder (47.9 mg, 98%) (Fig. S3). ^{1}H NMR (400 MHz, $CDCl_3$) δ 2.49 (q, J = 7.3 Hz, 16H), 0.31 (t, J = 7.3 Hz, 24H); ^{13}C NMR (100 MHz, $CDCl_3$) δ 140.6 (4°), 136.4 (4°), 130.5 (4°), 55.5 (4°), 26.3 (CH_2), 8.3 (CH_3); HRMS (ESI-TOF) *m*/*z*: $[M+Na]^+$ calcd for $C_{44}H_{48}O_8Na$ 727.3241; found 727.3258.

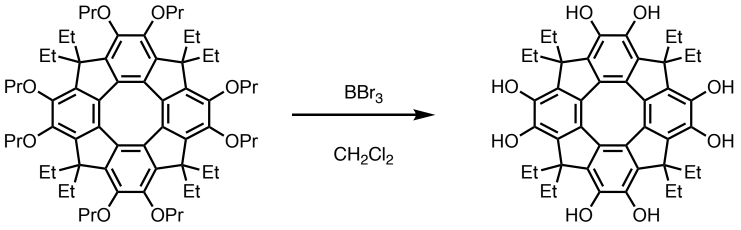

Fig. S3. The synthetic scheme of **1**.

Synthesis of **TCTP-COF**

To a 6-mL glass tube were added **1** (19.5 mg, 28.4 μmol), $B(OMe)_3$ (6.3 μL, 57 μmol) and *N*,*N*-dibutylformamide (DBF; 0.93 mL) in an argon filled glove box. The mixture was degassed by freeze-pump-thaw cycling and sealed under vacuum using a Schlenk line and oil pump. After warming to room temperature, the reaction tube was put into an oven at 120 °C for 3 days. After cooling to ambient temperature, the crude product was washed with hexane, EtOAc, water, $CHCl_3$ and Et_2O and then dried *in vacuo* to afford **TCTP-COF** as pale blue crystalline solid (16.5 mg, 61%) (Fig. S4).

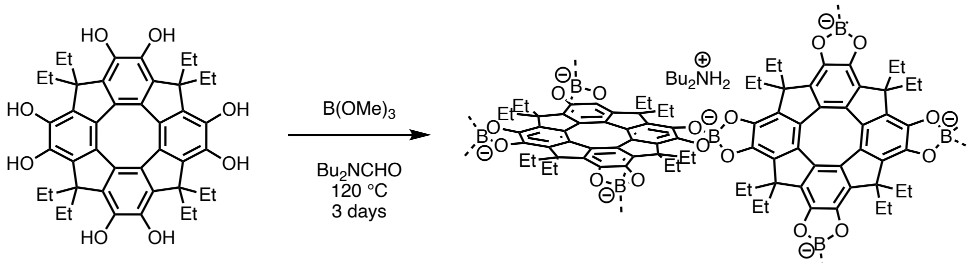

1 **TCTP-COF**

Fig. S4. The synthetic scheme of **TCTP-COF**.

Synthesis of **TCTP-COF**

To a solution of catechol (125 mg, 1.14 mmol) in DMF (6.0 mL) was added trimethyl borate (64.0 μL, 568 μmol, 0.5 equiv). The reaction mixture was stirred at 120 °C for 3 days. After completion, the mixture was concentrated under reduced pressure. The residue was reprecipitated with dichloromethane and diethyl ether, and the resulting solid was washed with diethyl ether and dried *in vacuo* to afford **6** (69.9 mg, 17% yield) (Fig. S5). The ^{1}H and ^{13}C NMR spectra was consistent with the reported ones (*25*).

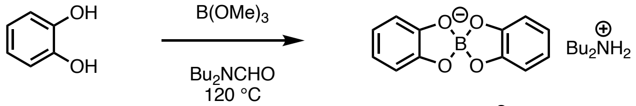

6

Fig. S5. The synthetic scheme of **6**.

3. Supplementary figures and tables

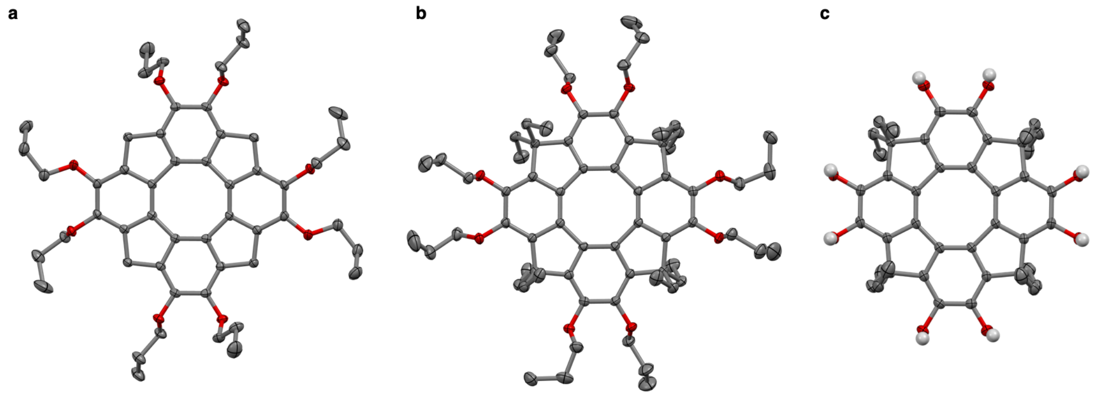

Fig. S6. Molecular structures of **4** (a), **5** (b), and **1** (c) with thermal ellipsoids at 50% probability. Solvent molecules, the minor parts of disordered moieties, and hydrogen atoms except OH were omitted for clarity.

Table S1. Crystallographic data and structure refinement details of **4**, **5**, and **1**.

	**4**	**5**	**1**
CCDC No.	2483958	2483959	2483960
formula	$C_{52}H_{64}O_8$	$C_{68}H_{96}O_8$	$C_{23}H_{28}O_5$
fw	817.03	1041.44	384.45
T (K)	143.15	143.18(10)	143.15
λ (Å)	0.71073	1.54184	0.71073
cryst syst	monoclinic	monoclinic	monoclinic
space group	$P2_1/n$	$C2/c$	$C2/c$
a (Å)	4.6835(2)	27.3010(9)	16.7383(13)
b (Å)	17.2972(6)	14.1767(5)	12.0461(11)
c (Å)	26.9516(12)	16.4244(5)	24.0879(18)
α (deg)	90	90	90
β (deg)	91.305(4)	105.975(4)	102.759(8)
γ (deg)	90	90	90
V (Å^3)	2182.82(15)	6111.4(4)	4736.9(7)
Z	2	4	8
D_{calc} (g·cm^{-3})	1.243	1.132	1.078
μ (mm^{-1})	0.082	0.563	0.075
F(000)	880.0	2272.0	1648.0
cryst size (mm)	0.10 × 0.10 × 0.03	0.05 × 0.05 × 0.03	0.10 × 0.01 × 0.01
2θ range (deg)	5.11–63.558	6.736–143.234	5.412–62.67
reflns collected	16612	12952	24426
indep reflns/R_{int}	5960 / 0.0620	5810 / 0.0293	6575 / 0.0859
params	342	363	258
GOF on F^2	1.052	1.047	1.082
R_1, wR_2 [I>2$\sigma(I)$]	0.0622, 0.1463	0.0566, 0.1525	0.0790, 0.2507
R_1, wR_2 (all data)	0.1073, 0.1667	0.0666, 0.1630	0.1118, 0.2694

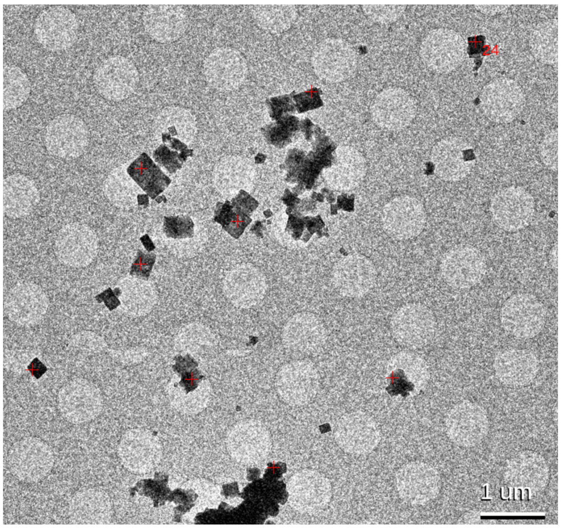

Fig. S7. An electron microscope image of **TCTP-COF** on a MicroED grid at a x2600 magnification. Discontinuities in the image are due to errors in the montage alignment.

Table S2. Crystallographic data and structure refinement details of **TCTP-COF**.

	**TCTP-COF**
CCDC No.	2490535
COD No.	3000619
formula	$C_{22}H_{20}BO_4$
fw	359.21
T (K)	96
λ (Å)	0.02508
cryst syst	cubic
space group	*Im*-3*m*
a (Å)	25.2887(3)
V (Å^3)	16172.6(3)
Z	12
reflns collected	1607590
indep reflns/R_{int}/R_{sigma}	1746 / 0.4013 / 0.0258
Data/restraints/parameters	1746 / 0 / 44
GOF on F^2	1.382
R_1, wR_2 [I>2σ(I)]	0.1256, 0.3357
maximum resolution used for refinement (Å)	0.7804

Table S3. Merging statistics of the **TCTP-COF** MicroED dataset.

d_max	d_min	#obs	#uniq	mult.	%comp	<I/sI>	r_pim	cc1/2
6.32	2.09	59127	107	552.6	100	112.0	0.009	0.993*
2.09	1.67	77208	96	804.3	100	92.6	0.010	0.999*
1.67	1.46	81447	94	866.5	100	65.4	0.011	0.998*
1.46	1.33	78750	90	875.0	100	43.4	0.015	0.994*
1.33	1.24	80844	89	908.4	100	39.5	0.014	1.000*
1.24	1.16	79334	85	933.3	100	31.8	0.016	0.999*
1.16	1.11	80543	87	925.8	100	40.6	0.013	0.999*
1.11	1.06	83581	86	971.9	100	28.7	0.016	0.999*
1.06	1.02	80014	88	909.3	100	19.3	0.022	0.997*
1.02	0.98	82756	85	973.6	100	11.0	0.028	0.997*
0.98	0.95	74546	77	968.1	100	8.3	0.035	0.981*
0.95	0.92	88272	92	959.5	100	5.4	0.046	0.974*
0.92	0.90	79819	82	973.4	100	4.0	0.061	0.971*
0.90	0.88	81669	84	972.3	100	3.6	0.062	0.975*
0.88	0.86	86333	87	992.3	100	2.5	0.085	0.902*
0.86	0.84	75240	78	964.6	100	2.2	0.092	0.922*
0.84	0.82	86567	86	1006.6	100	1.6	0.100	0.607*
0.82	0.81	81126	83	977.4	100	1.7	0.102	0.770*
0.81	0.79	83512	83	1006.2	100	1.1	0.149	0.521*
0.79	0.78	87331	87	1003.8	100	1.3	0.129	0.658*
6.32	0.78	1608019	1746	921.0	100	27.7	0.014	0.995*

Table S4. Fractional atomic coordinates for **TCTP-COF** obtained from Rietveld refinement.

	x	*y*	*z*	U_{iso}	U_{11}	U_{22}	U_{33}	U_{12}	U_{13}	U_{23}
C	1.00000	0.52797	0.43135		0.04190	0.03020	0.02200	0.00000	0.00000	0.00420
C	1.00000	0.52758	0.33486		0.06500	0.02640	0.02910	0.00000	0.00000	0.00710
O	1.00000	0.54617	0.28544		0.06850	0.05690	0.02580	0.00000	0.00000	-0.00170
C	1.00000	0.55500	0.38091		0.07000	0.02970	0.01810	0.00000	0.00000	0.00070
B	1.00000	0.50000	0.25000		0.03700	0.03700	0.02400	0.00000	0.00000	0.00000
C	1.00000	0.61371	0.38629		0.07000	0.02890	0.02890	0.00000	0.00000	-0.00120
C	0.95035	0.63903	0.36097		0.08100	0.04090	0.04090	0.01270	-0.01270	0.00000
H	0.95135	0.67770	0.36701	0.06500						
H	0.95135	0.63299	0.32230	0.06500						
C	0.90010	0.61782	0.38218		0.08200	0.08800	0.08800	0.02440	0.02440	0.00800
H	0.87030	0.63532	0.36468	0.12900						
H	0.89840	0.57968	0.37558	0.12900						
H	0.89840	0.62442	0.42032	0.12900						

Space Group: *Im*-3*m*; Unit cell: $a = b = c = 25.2830(17)$, $\alpha = \beta = \gamma = 90°$, $V = 16162(3)$ Å^3; $wR = 5.80\%$, chi**2 = 5981.18, Reduced $X^2 = 1.27$, GOF = 1.13

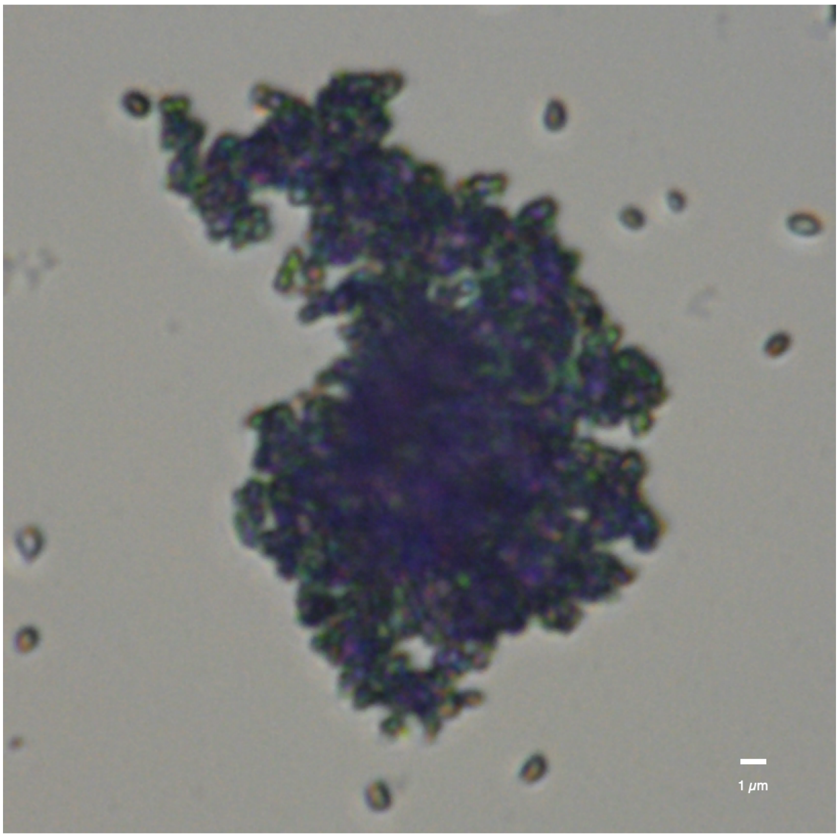

Fig. S8. Microscope image of the crystals of **TCTP-COF**.

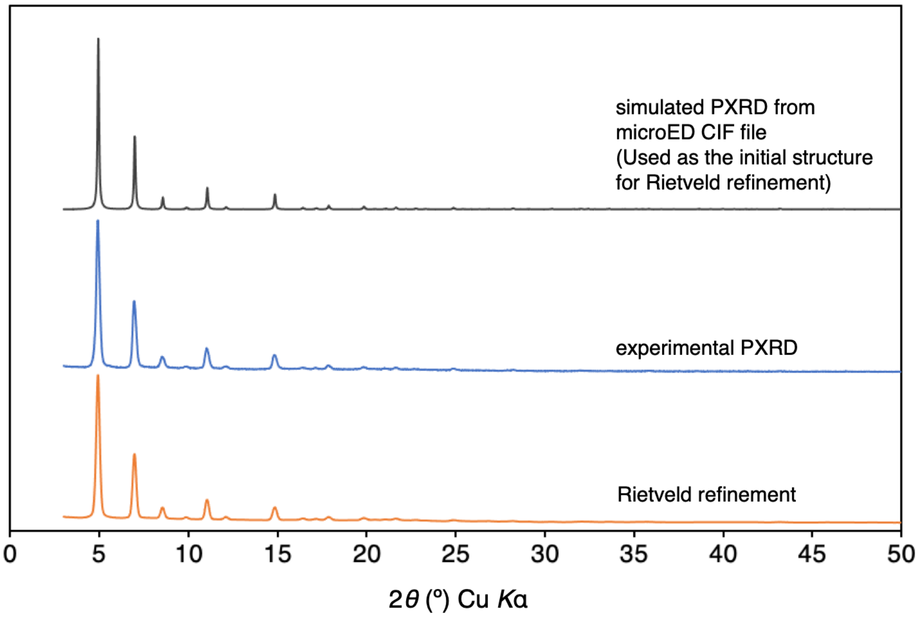

Fig. S9. The experimental PXRD pattern and Rietveld refinement of **TCTP-COF** compared with the simulated PXRD pattern generated from the CIF file obtained by microED. Mercury 4.2.0 was used for PXRD simulation with the full width at half maximum (FXHM) value of 0.1.

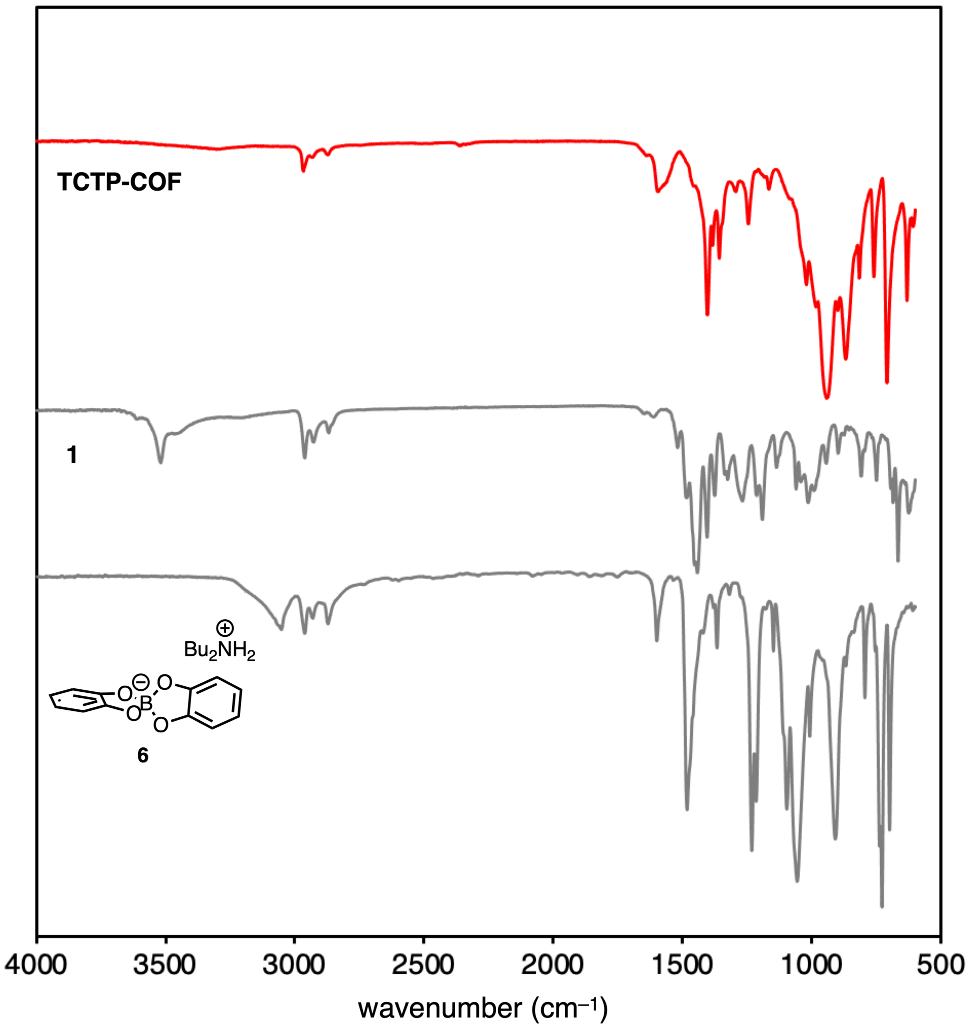

Fig. S10. FT-IR spectra of **TCTP-COF**, **1**, and a model compound **6**.

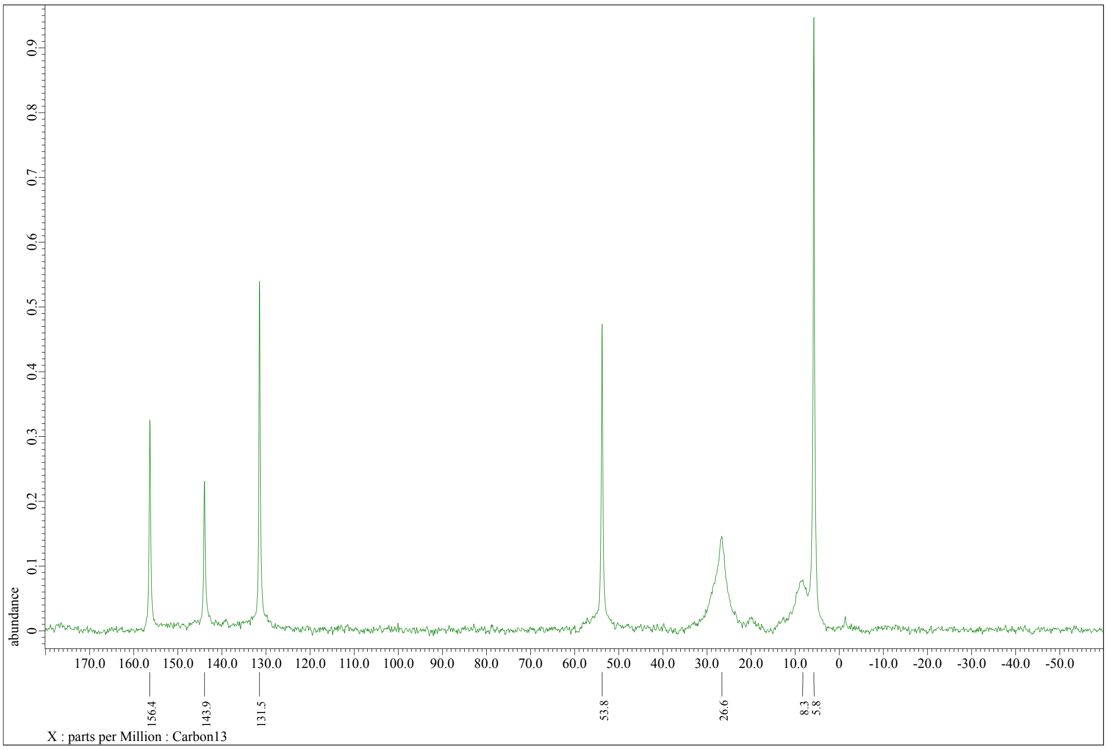

Fig. S11. Solid state ^{13}C NMR spectrum of **TCTP-COF**.

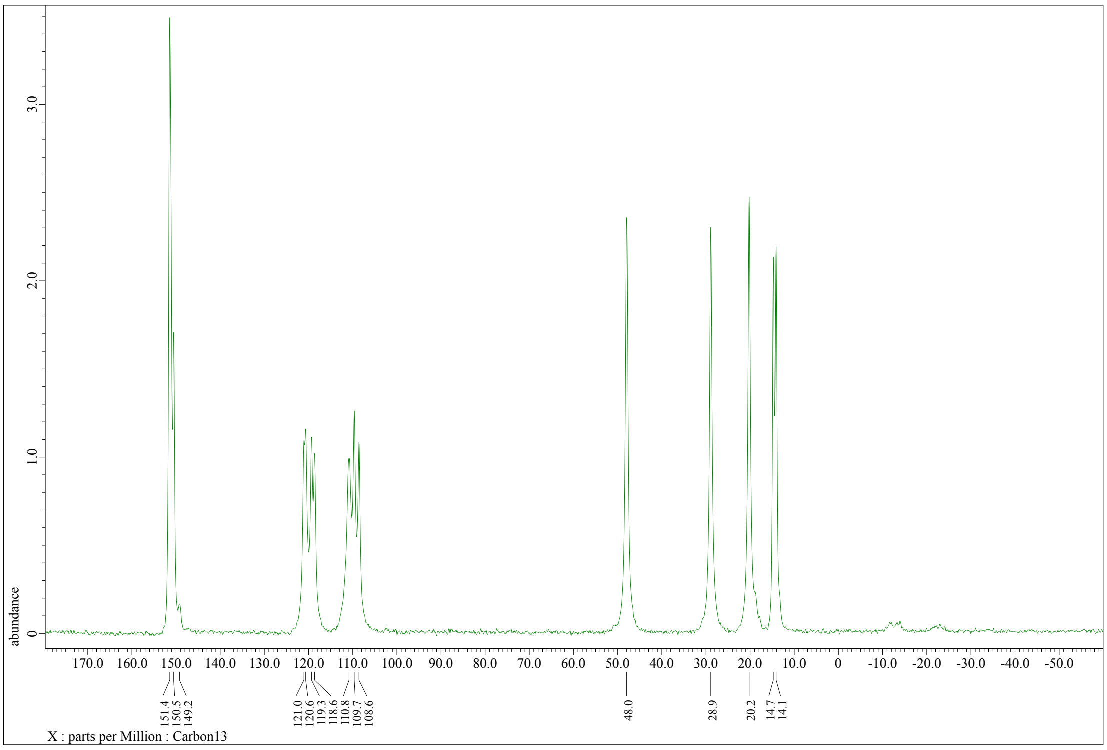

Fig. S12. Solid state ^{13}C NMR spectrum of **6**.

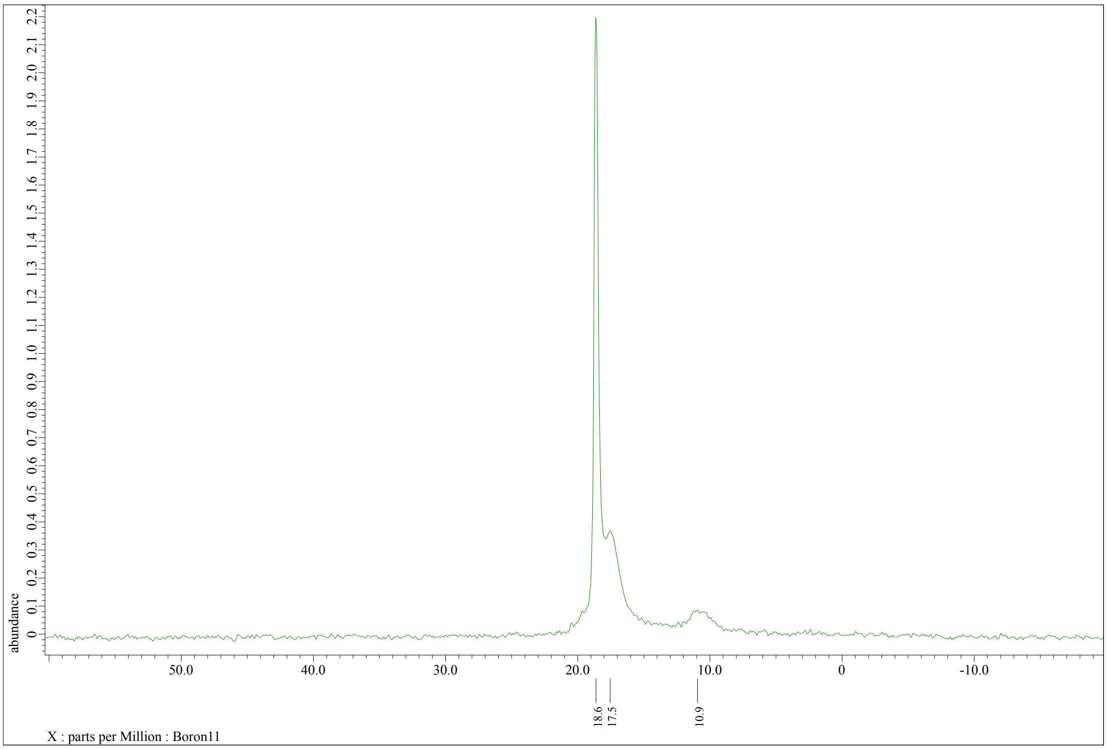

Fig. S13. Solid state ^{11}B NMR spectrum of **TCTP-COF**.

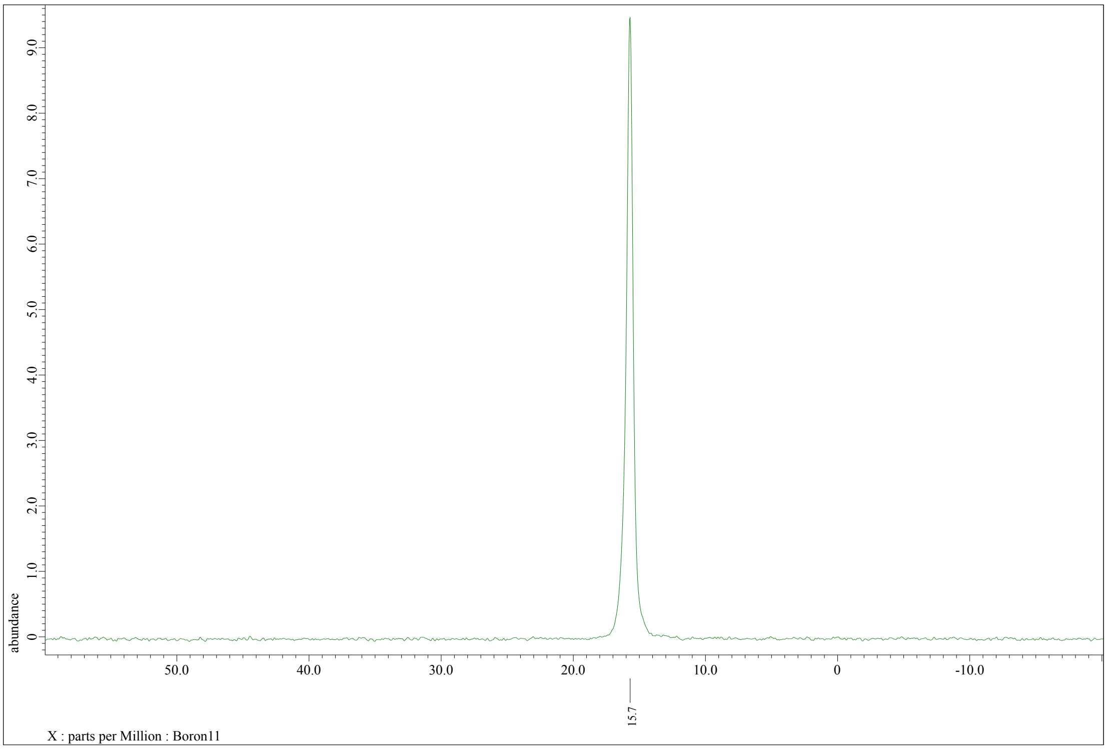

Fig. S14. Solid state ^{11}B NMR spectrum of **6**.

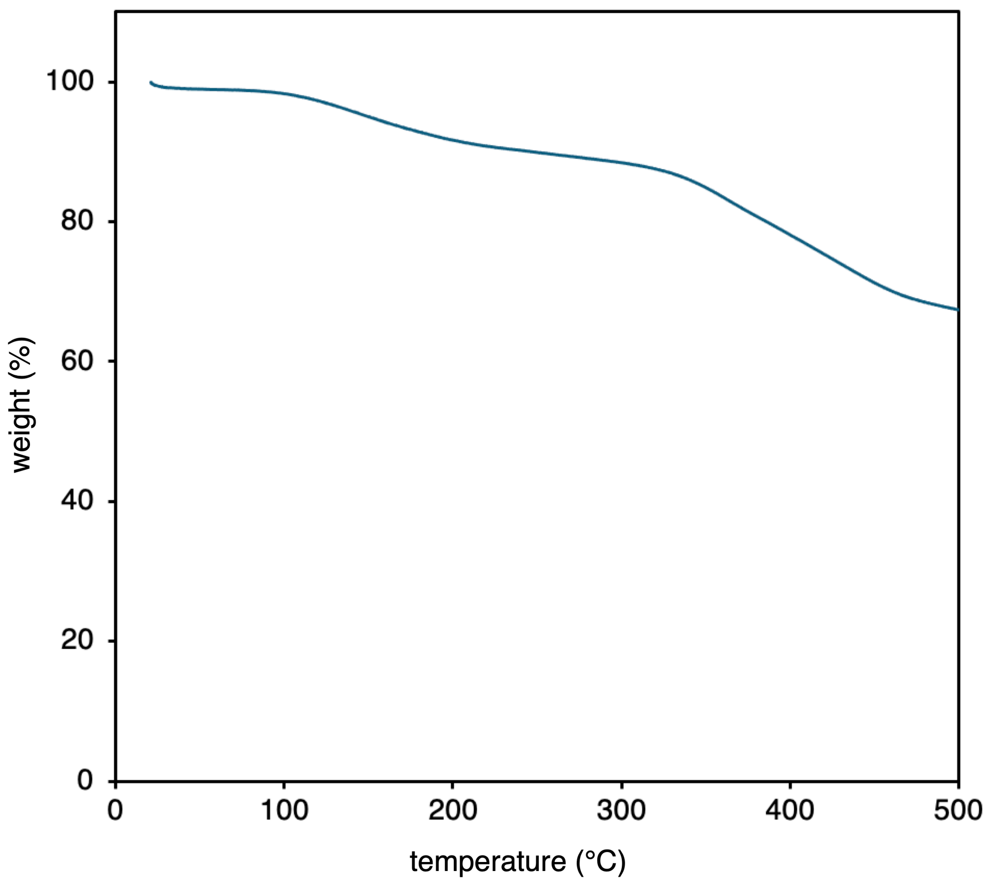

Fig. S15. TGA curves of **TCTP-COF** in N_2 atmosphere.

Table S5. Electronic energies (E) of stationary points (Hartree).

compound	E	compound	E
**TCTP (120°)**	-3128.71483641	**Pc (120°)**	-4440.97742528
**TCTP (123°)**	-3128.71736236	**Pc (123°)**	-4440.97922791
**TCTP (126°)**	-3128.71972041	**Pc (126°)**	-4440.98092304
**TCTP (129°)**	-3128.72191572	**Pc (129°)**	-4440.98251257
**TCTP (132°)**	-3128.72395363	**Pc (132°)**	-4440.98400074
**TCTP (135°)**	-3128.72584030	**Pc (135°)**	-4440.98539186
**TCTP (138°)**	-3128.72758220	**Pc (138°)**	-4440.98668581
**TCTP (141°)**	-3128.72918260	**Pc (141°)**	-4440.98788066
**TCTP (144°)**	-3128.73064428	**Pc (144°)**	-4440.98897695
**TCTP (147°)**	-3128.73197080	**Pc (147°)**	-4440.98997785
**TCTP (150°)**	-3128.73316814	**Pc (150°)**	-4440.99088614
**TCTP (153°)**	-3128.73424178	**Pc (153°)**	-4440.99170246
**TCTP (156°)**	-3128.73519554	**Pc (156°)**	-4440.99243801
**TCTP (159°)**	-3128.73603058	**Pc (159°)**	-4440.99308331
**TCTP (162°)**	-3128.73674678	**Pc (162°)**	-4440.99363829
**TCTP (165°)**	-3128.73734551	**Pc (165°)**	-4440.99410358
**TCTP (168°)**	-3128.73782921	**Pc (168°)**	-4440.99448196
**TCTP (171°)**	-3128.73819987	**Pc (171°)**	-4440.99477789
**TCTP (174°)**	-3128.73845816	**Pc (174°)**	-4440.99499814
**TCTP (177°)**	-3128.73860445	**Pc (177°)**	-4440.99513740
**TCTP (180°)**	-3128.73863916	**Pc (180°)**	-4440.99516887

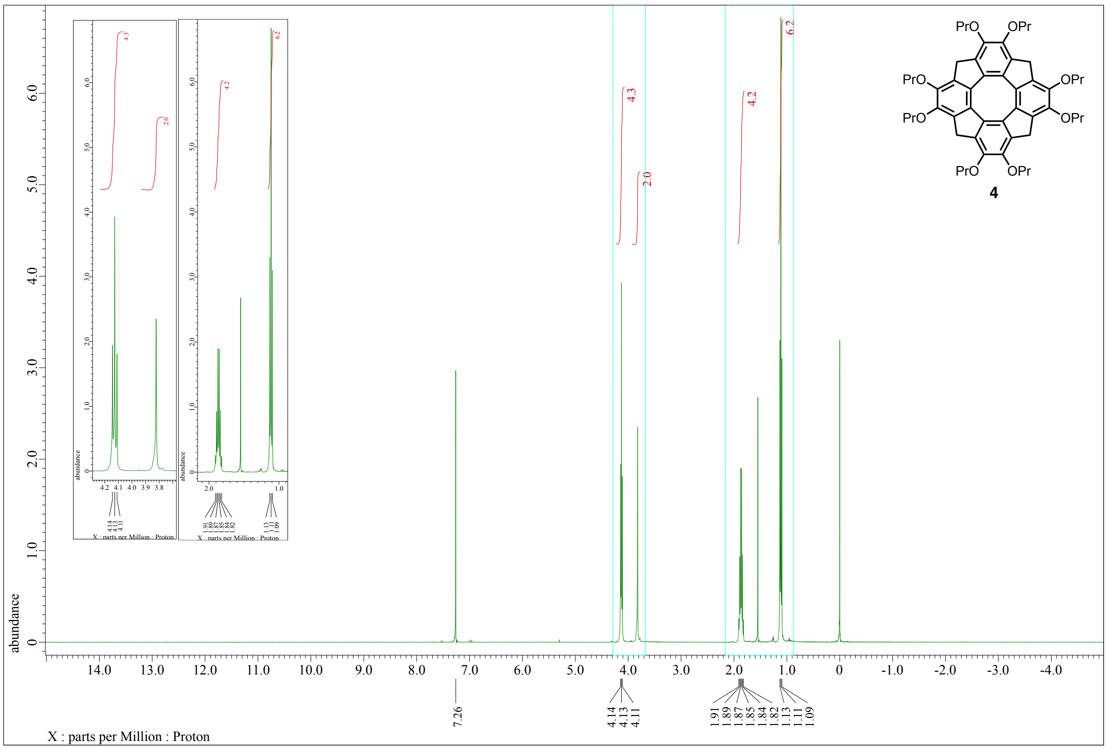

Fig. S16. ^{1}H NMR spectrum of **4** (400 MHz, $CDCl_3$)

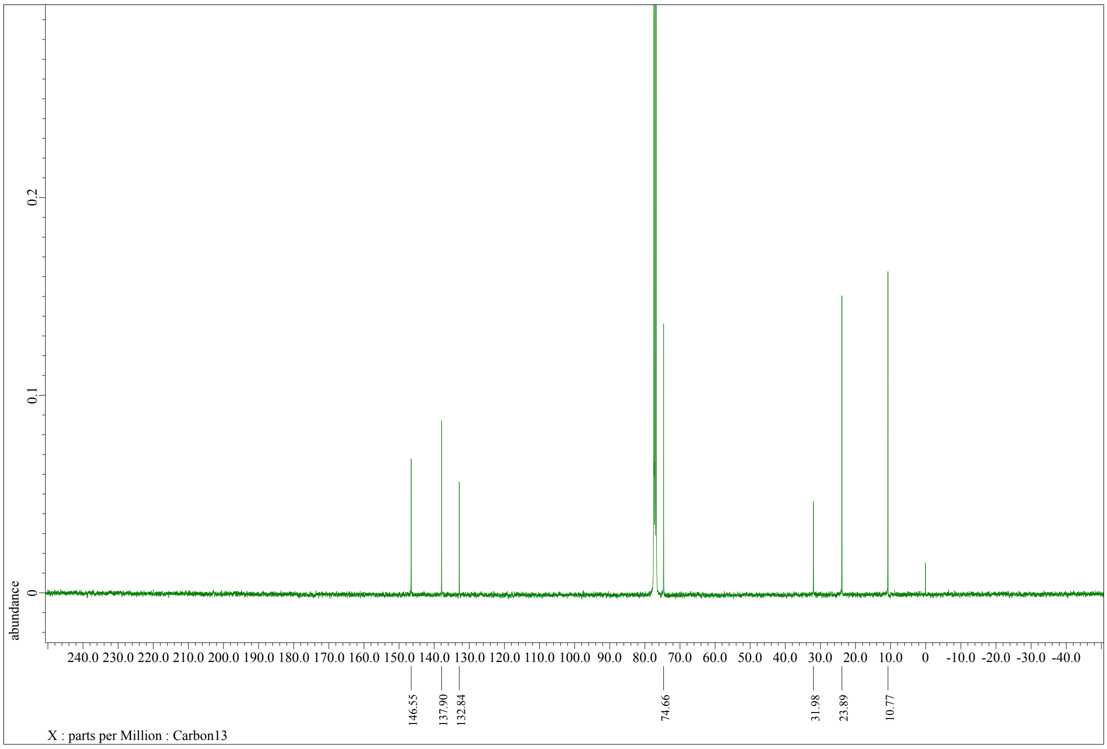

Fig. S17. ^{13}C NMR spectrum of **4** (100 MHz, $CDCl_3$)

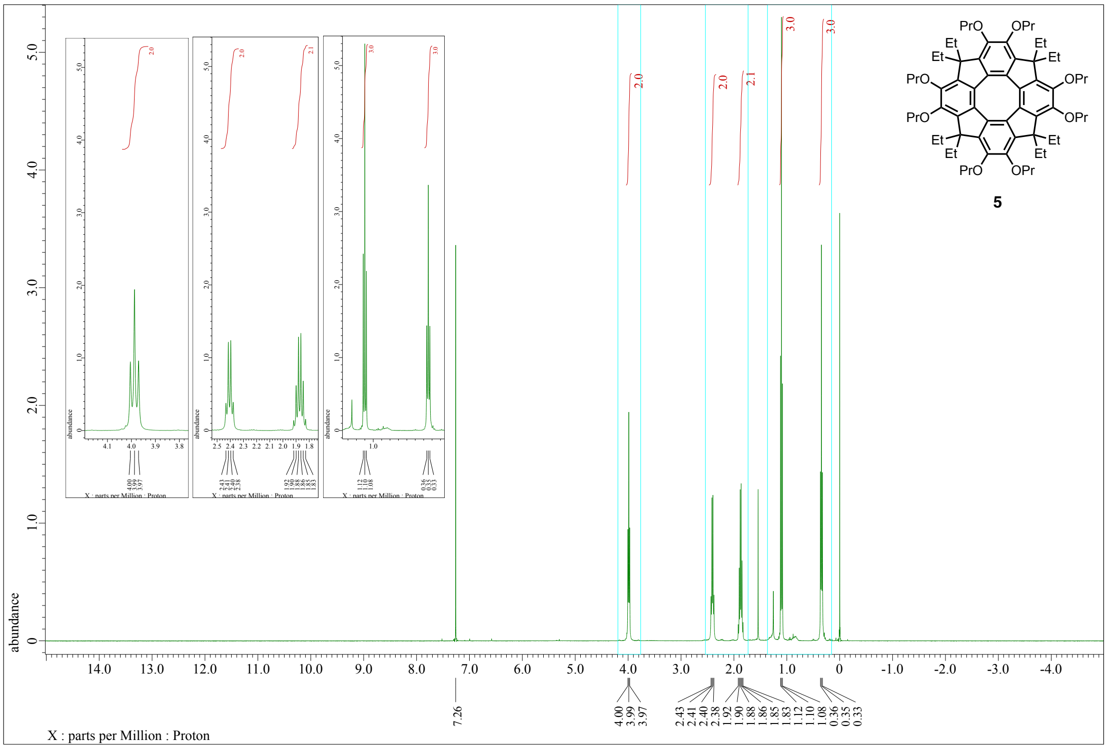

Fig. S18. ^{1}H NMR spectrum of **5** (400 MHz, $CDCl_3$)

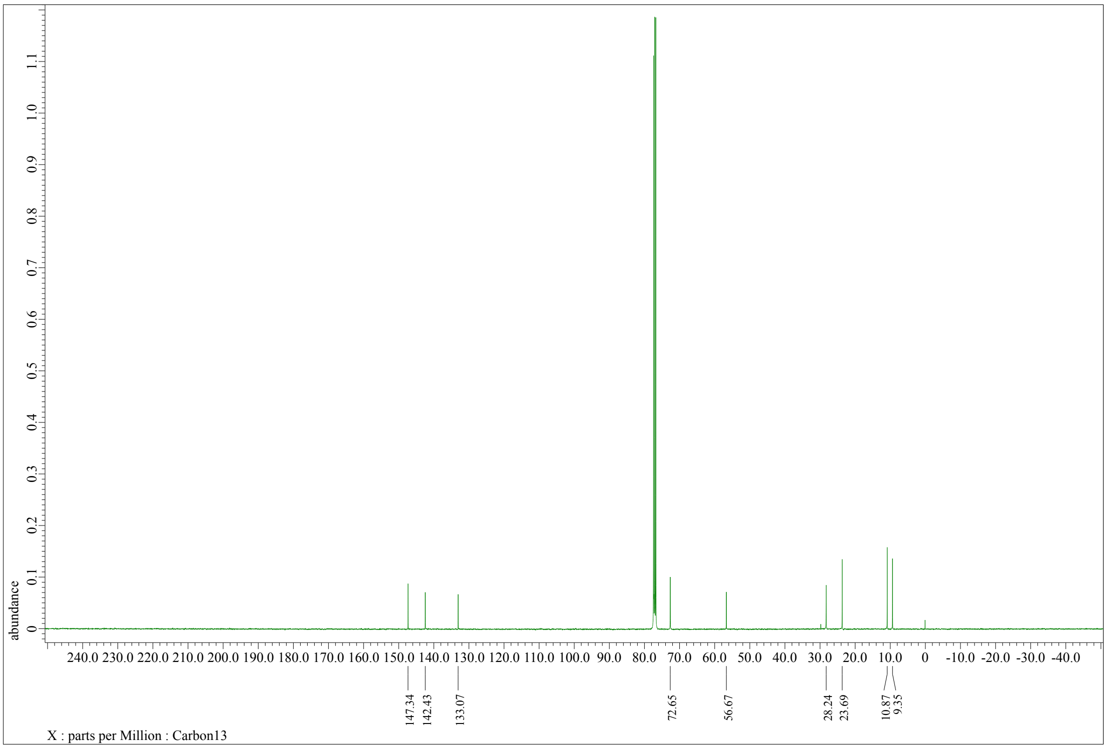

Fig. S19. ^{13}C NMR spectrum of **5** (100 MHz, $CDCl_3$)

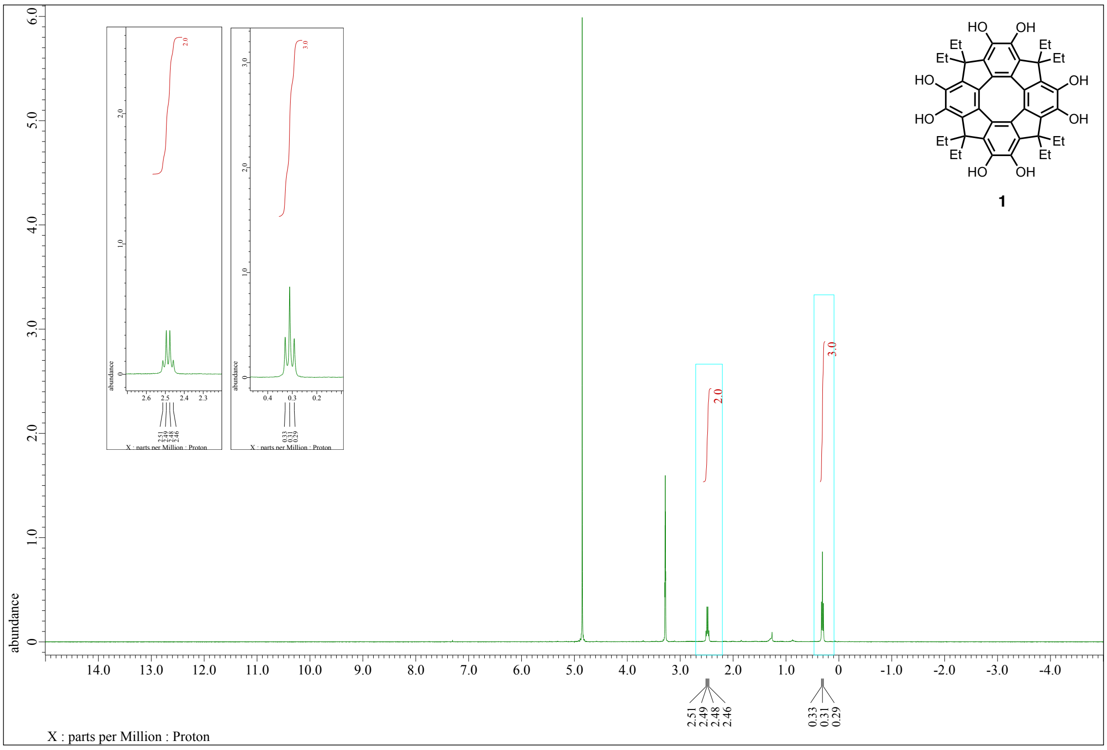

Fig. S20. ^{1}H NMR spectrum of **1** (400 MHz, $CDCl_3$)

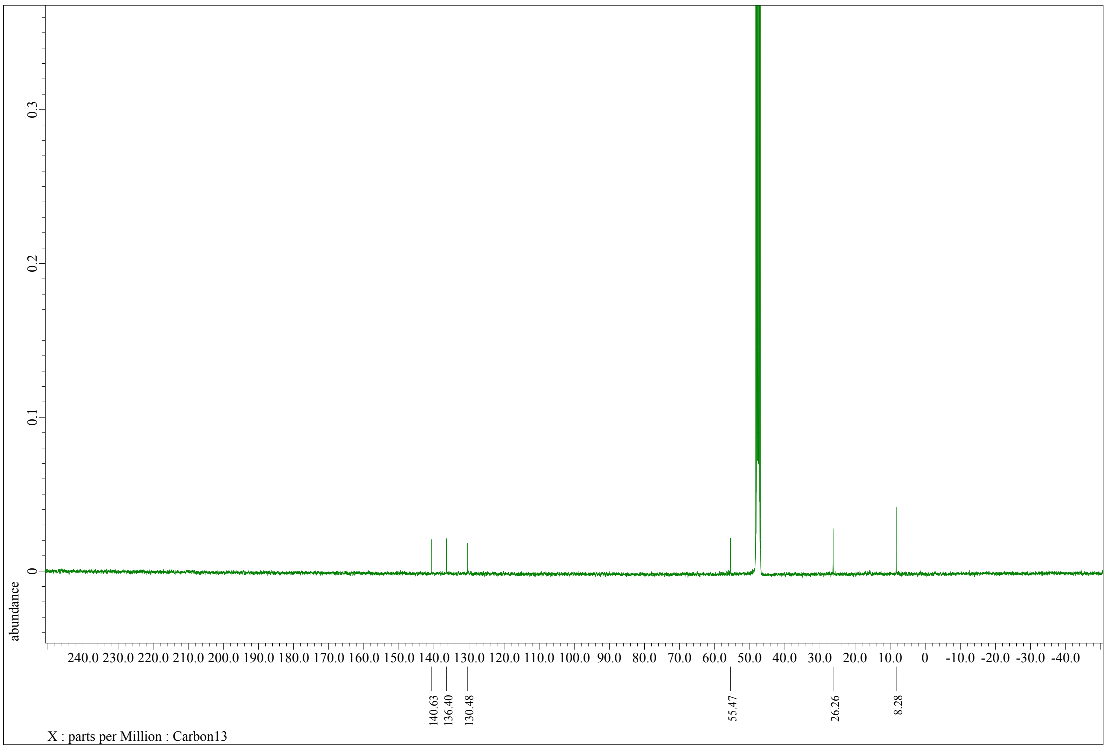

Fig. S21. ^{13}C NMR spectrum of **1** (100 MHz, $CDCl_3$)

REFERENCES

1. C. S. Diercks, O. M. Yaghi, The atom, the molecule, and the covalent organic framework. *Science* **355**, eaal1585 (2017).

2. R. Liu, K. T. Tan, Y. Gong, Y. Chen, Z. Li, S. Xie, T. He, Z. Lu, H. Yang, D. Jiang, Covalent organic frameworks: An ideal platform for designing ordered materials and advanced applications. *Chem. Soc. Rev.* **50**, 120–242 (2021).

3. K. Geng, T. He, R. Liu, S. Dalapati, K. T. Tan, Z. Li, S. Tao, Y. Gong, Q. Jiang, D. Jiang, Covalent organic frameworks: Design, synthesis, and functions. *Chem. Rev.* **120**, 8814–8933 (2020).

4. A. M. Evans, M. J. Strauss, A. R. Corcos, Z. Hirani, W. Ji, L. S. Hamachi, X. Aguilar-Enriquez, A. D. Chavez, B. J. Smith, W. R. Dichtel, Two-dimensional polymers and polymerizations. *Chem. Rev.* **122**, 442–564 (2022).

5. Y. Yusran, B. Miao, S. Qiu, Q. Fang, Functional covalent organic frameworks: Design principles to potential applications. *Acc. Mater. Res.* **5**, 1263–1278 (2024).

6. T. Kim, J. Kim, N. Kim, H. Lim, Recent advances in single crystal COFs from synthesis to applications. *Chem. Commun.* **61**, 17516–17529 (2025).

7. Y. Zeng, H. Zhang, M. Li, Z. Deng, Z. Li, J. Zhang, X. Chen, Y. Tian, Y. Chen, Frontiers in single-crystal covalent organic frameworks resolved by single crystal X-ray diffraction. *Inorg. Chem. Front.* **13**, 1291–1309 (2026).

8. T. Y. Lee, J. Yoon, E. Jo, S. Woo, K. Lee, S. Koo, D. W. Kang, Design and synthesis of single-crystalline covalent organic frameworks: The use of modulator for reversible bond control. *Chem. A Eur. J.* **31**, e01731 (2025).

9. C. Qian, H. Wu, W. L. Teo, Y. Liao, Y. Zhao, Single-crystalline covalent organic frameworks. *Trends Chem.* **5**, 853–867 (2023).

10. M. Gemmi, E. Mugnaioli, T. E. Gorelik, U. Kolb, L. Palatinus, P. Boullay, S. Hovmöller, J. P. Abrahams, 3D electron diffraction: The nanocrystallography revolution. *ACS Cent. Sci.* **5**, 1315–1329 (2019).

11. Z. Huang, E. S. Grape, J. Li, A. K. Inge, X. Zou, 3D electron diffraction as an important technique for structure elucidation of metal-organic frameworks and covalent organic frameworks. *Coord. Chem. Rev.* **427**, 213583 (2021).

12. Y.-B. Zhang, J. Su, H. Furukawa, Y. Yun, F. Gándara, A. Duong, X. Zou, O. M. Yaghi, Single-crystal structure of a covalent organic framework. *J. Am. Chem. Soc.* **135**, 16336–16339 (2013).

13. D. Beaudoin, T. Maris, J. D. Wuest, Constructing monocrystalline covalent organic networks by polymerization. *Nat. Chem.* **5**, 830–834 (2013).

14. T. Ma, E. A. Kapustin, S. X. Yin, L. Liang, Z. Zhou, J. Niu, L.-H. Li, Y. Wang, J. Su, J. Li, X. Wang, W. D. Wang, W. Wang, J. Sun, O. M. Yaghi, Single-crystal x-ray diffraction structures of covalent organic frameworks. *Science* **361**, 48–52 (2018).

15. A. Natraj, W. Ji, J. Xin, I. Castano, D. W. Burke, A. M. Evans, M. J. Strauss, M. Ateia, L. S. Hamachi, N. C. Gianneschi, Z. A. ALOthman, J. Sun, K. Yusuf, W. R. Dichtel, Single-crystalline imine-linked two-dimensional covalent organic frameworks separate benzene and cyclohexane efficiently. *J. Am. Chem. Soc.* **144**, 19813–19824 (2022).

16. B. Hou, X. Han, H. Xie, C. Yuan, Y. Guo, X. Chen, X. Tang, S. Su, H. Jiang, Z.-M. Ye, K. O. Kirlikovali, Y. Liu, O. K. Farha, Y. Cui, Single-crystal x-ray structures of homochiral brønsted acidic covalent organic frameworks. *J. Am. Chem. Soc.* **147**, 12127–12137 (2025).

17. F. Tan, S. Han, D. Peng, H. Wang, J. Yang, P. Zhao, X. Ye, X. Dong, Y. Zheng, N. Zheng, L. Gong, C. Liang, N. Frese, A. Gölzhäuser, H. Qi, S. Chen, W. Liu, Z. Zheng, Nanoporous and highly thermal conductive thin film of single-crystal covalent organic frameworks ribbons. *J. Am. Chem. Soc.* **143**, 3927–3933 (2021).

18. M. Wang, Y. Jin, W. Zhang, Y. Zhao, Single-crystal polymers (SCPs): From 1D to 3D architectures. *Chem. Soc. Rev.* **52**, 8165–8193 (2023).

19. Y. Hu, S. J. Teat, W. Gong, Z. Zhou, Y. Jin, H. Chen, J. Wu, Y. Cui, T. Jiang, X. Cheng, W. Zhang, Single crystals of mechanically entwined helical covalent polymers. *Nat. Chem.* **13**, 660–665 (2021).

20. Y. Hu, N. Dunlop, H. Long, H. Chen, L. Wayment, M. Ortiz, Y. Jin, A. Nijamudheen, J. Mendoza-Cortes, S.-H. Lee, W. Zhang, Helical covalent polymers with unidirectional ion channels as single lithium-ion conducting electrolytes. *CCS Chem.* **3**, 2762–2770 (2021).

21. L. J. Wayment, S. Huang, H. Chen, Z. Lei, A. Ley, S.-H. Lee, W. Zhang, Ionic covalent organic frameworks consisting of tetraborate nodes and flexible linkers. *Angew. Chem. Int. Ed.* **63**, e202410816 (2024).

22. S. Xue, Q. Wei, R. Zhang, T. Zhang, G. Duan, X. Han, K. Liu, J. Han, S. He, S. Jiang, Spiroborate-based three dimensional covalent organic framework for effective adsorption and separation of organic dyes. *Sep. Purif. Technol.* **341**, 126941 (2024).

23. L. J. Wayment, X. Wang, S. Huang, M. S. McCoy, H. Chen, Y. Hu, Y. Jin, S. Sharma, W. Zhang, 3D covalent organic framework as a metastable intermediate in the formation of a double-stranded helical covalent polymer. *J. Am. Chem. Soc.* **145**, 15547–15552 (2023).

24. H. Chen, Y. Hu, C. Luo, Z. Lei, S. Huang, J. Wu, Y. Jin, K. Yu, W. Zhang, Spiroborate-linked ionic covalent adaptable networks with rapid reprocessability and closed-loop recyclability. *J. Am. Chem. Soc.* **145**, 9112–9117 (2023).

25. X. Wang, M. Bahri, Z. Fu, M. A. Little, L. Liu, H. Niu, N. D. Browning, S. Y. Chong, L. Chen, J. W. Ward, A. I. Cooper, A cubic 3D covalent organic framework with nbo topology. *J. Am. Chem. Soc.* **143**, 15011–15016 (2021).

26. X. Wang, T. Fellowes, M. Bahri, H. Qu, B. Li, H. Niu, N. D. Browning, W. Zhang, J. W. Ward, A. I. Cooper, 2D to 3D reconstruction of boron-linked covalent–organic frameworks. *J. Am. Chem. Soc.* **146**, 14128–14135 (2024).

27. X. Wang, Q. Zhu, H. Qu, X. Zhou, M. Bahri, B. Liu, T. Fellowes, R. Clowes, H. Niu, N. D. Browning, A. I. Cooper, Influence of methyl substitution on linear diboronic acids: Toward spiroborate covalent organic framework formation in *N*, *N*-diethylformamide. *J. Mater. Chem. A* **13**, 19374–19380 (2025).

28. D. Hellwinkel, G. Reiff, V. Nykodym, Verbrückte tetraphenylen-systeme. *Justus Liebigs Ann. Chem.* **1977**, 1013–1025 (1977).

29. S. Nobusue, H. Miyoshi, A. Shimizu, I. Hisaki, K. Fukuda, M. Nakano, Y. Tobe, Tetracyclopenta[*def,jkl,pqr,vwx*]tetraphenylene: A potential tetraradicaloid hydrocarbon. *Angew. Chem. Int. Ed.* **54**, 2090–2094 (2015).

30. S. Hirota, S. Nakano, H. Sugiyama, Y. Segawa, Synthesis of polycyclic arenes composed of four-, five-, six-, and eight-membered rings via an unexpected four-membered ring formation reaction. *Org. Lett.* **25**, 8062–8066 (2023).

31. A. Matsuo, T. Tanaka, O. Yusuke, Scholl reaction of ortho-phenylene-bridged cyclic pyrrole-thiophene hybrid hexamer. *Synthesis* **54**, 147–152 (2021).

32. T. Hensel, N. N. Andersen, M. Plesner, M. Pittelkow, Synthesis of heterocyclic [8]circulenes and related structures. *Synlett* **27**, 498–525 (2016).

33. Y. Miyake, H. Shinokubo, Hetero[8]circulenes: Synthetic progress and intrinsic properties. *Chem. Commun.* **56**, 15605–15614 (2020).

34. P. Schneider, Adsorption isotherms of microporous-mesoporous solids revisited. *Appl. Catal. Gen.* **129**, 157–165 (1995).

35. G. M. Sheldrick, Crystal structure refinement with SHELXL. *Acta Crystallogr. C Struct. Chem.* **71**, 3–8 (2015).

36. G. M. Sheldrick, SHELXT–Integrated space-group and crystal-structure determination. *Acta Crystallogr. A Found. Crystallogr.* **71**, 3–8 (2015).

37. O. V. Dolomanov, L. J. Bourhis, R. J. Gildea, J. A. K. Howard, H. Puschmann, OLEX2: A complete structure solution, refinement and analysis program. *J. Appl. Cryst.* **42**, 339–341 (2009).

38. J.-S. Jiang, A. T. Brünger, Protein hydration observed by X-ray diffraction: Solvation properties of penicillopepsin and neuraminidase crystal structures. *J. Mol. Biol.* **243**, 100–115 (1994).

39. D. N. Mastronarde, SerialEM: A program for automated tilt series acquisition on tecnai microscopes using prediction of specimen position. *Microsc. Microanal.* **9**, 1182–1183 (2003).

40. H. Hamada, T. Nakamuro, K. Yamashita, H. Yanagisawa, O. Nureki, M. Kikkawa, K. Harano, R. Shang, E. Nakamura, Spiro-conjugated carbon/heteroatom-bridged p-phenylenevinylenes: Synthesis, properties, and microcrystal electron crystallographic analysis of racemic solid solutions. *Bull. Chem. Soc. Jpn.* **93**, 776–782 (2020).

41. H. Lu, T. Nakamuro, K. Yamashita, H. Yanagisawa, O. Nureki, M. Kikkawa, H. Gao, J. Tian, R. Shang, E. Nakamura, B/N-doped *p*-arylenevinylene chromophores: Synthesis, properties, and microcrystal electron crystallographic study. *J. Am. Chem. Soc.* **142**, 18990–18996 (2020).

42. J. Hattne, M. W. Martynowycz, P. A. Penczek, T. Gonen, MicroED with the Falcon III direct electron detector. *IUCrJ* **6**, 921–926 (2019).

43. G. Winter, D. G. Waterman, J. M. Parkhurst, A. S. Brewster, R. J. Gildea, M. Gerstel, L. Fuentes-Montero, M. Vollmar, T. Michels-Clark, I. D. Young, N. K. Sauter, G. Evans, DIALS: Implementation and evaluation of a new integration package. *Acta Crystallogr. D Struct. Biol.* **74**, 85–97 (2018).

44. M. T. B. Clabbers, T. Gruene, J. M. Parkhurst, J. P. Abrahams, D. G. Waterman, Electron diffraction data processing with DIALS. *Acta Crystallogr. D Struct. Biol.* **74**, 506–518 (2018).

45. D. Gogoi, T. Sasaki, T. Nakane, A. Kawamoto, H. Hojo, G. Kurisu, R. Thakuria, Structure elucidation of olanzapine molecular salts by combining mechanochemistry and micro-electron diffraction. *Cryst. Growth Des.* **23**, 5821–5826 (2023).

46. O. Tange, GNU parallel: The command-line power tool. *login Usenix Mag.* **36**, 42–47 (2011).

47. R. J. Gildea, J. Beilsten-Edmands, D. Axford, S. Horrell, P. Aller, J. Sandy, J. Sanchez-Weatherby, C. D. Owen, P. Lukacik, C. Strain-Damerell, R. L. Owen, M. A. Walsh, G. Winter, xia2.multiplex: A multi-crystal data-analysis pipeline. *Acta Crystallogr. D Struct. Biol.* **78**, 752–769 (2022).

48. J. Beilsten-Edmands, G. Winter, R. Gildea, J. Parkhurst, D. Waterman, G. Evans, Scaling diffraction data in the DIALS software package: Algorithms and new approaches for multi-crystal scaling. *Acta Crystallogr. D Struct. Biol.* **76**, 385–399 (2020).

49. J. Schaefer, E. O. Stejskal, Carbon-13 nuclear magnetic resonance of polymers spinning at the magic angle. *J. Am. Chem. Soc.* **98**, 1031–1032 (1976).

50. A. E. Bennett, C. M. Rienstra, M. Auger, K. V. Lakshmi, R. G. Griffin, Heteronuclear decoupling in rotating solids. *J. Chem. Phys.* **103**, 6951–6958 (1995).

51. G. Metz, X. L. Wu, S. O. Smith, Ramped-amplitude cross polarization in magic-angle-spinning nmr. *J. Magn. Reson. Ser. A* **110**, 219–227 (1994).

52. M. J. Frisch, G. W. Trucks, H. B. Schlegel, G. E. Scuseria, M. A. Robb, J. R. Cheeseman, G. Scalmani, V. Barone, G. A. Petersson, H. Nakatsuji, X. Li, M. Caricato, A. V. Marenich, J. Bloino, B. G. Janesko, R. Gomperts, B. Mennucci, H. P. Hratchian, J. V. Ortiz, A. F. Izmaylov, J. L. Sonnenberg, D. Williams-Young, F. Ding, F. Lipparini, F. Egidi, J. Goings, B. Peng, A. Petrone, T. Henderson, D. Ranasinghe, V. G. Zakrzewski, J. Gao, N. Rega, G. Zheng, W. Liang, M. Hada, M. Ehara, K. Toyota, R. Fukuda, J. Hasegawa, M. Ishida, T. Nakajima, Y. Honda, O. Kitao, H. Nakai, T. Vreven, K. Throssell, J. A. Montgomery, Jr., J. E. Peralta, F. Ogliaro, M. J. Bearpark, J. J. Heyd, E. N. Brothers, K. N. Kudin, V. N. Staroverov, T. A. Keith, R. Kobayashi, J. Normand, K. Raghavachari, A. P. Rendell, J. C. Burant, S. S. Iyengar, J. Tomasi, M. Cossi, J. M. Millam, M. Klene, C. Adamo, R. Cammi, J. W. Ochterski,

R. L. Martin, K. Morokuma, O. Farkas, J. B. Foresman, and D. J. Fox, Gaussian 16, Revision C.02 (Gaussian, 2019).

53. A. D. Becke, Density-functional thermochemistry. III. the role of exact exchange. *J. Chem. Phys.* **98**, 5648–5652 (1993).

54. C. Lee, W. Yang, R. G. Parr, Development of the colle-salvetti correlation-energy formula into a functional of the electron density. *Phys. Rev. B.* **37**, 785–789 (1988).

55. W. C. Spaller, J. Q. Lu, B. J. Stokes, Tetrahydroxydiboron-mediated palladium-catalyzed deoxygenative transfer hydrogenation of aryl ketones. *Adv. Synth. Catal.* **364**, 2571–2575 (2022).
